# An update on evolutionary, structural, and functional studies of receptor-like kinases in plants

**DOI:** 10.3389/fpls.2024.1305599

**Published:** 2024-01-31

**Authors:** Jing Liu, Wenjuan Li, Guang Wu, Khawar Ali

**Affiliations:** College of Life Sciences, Shaanxi Normal University, Xi’an, China

**Keywords:** receptor-like kinases, evolution, phosphorylation, signaling, ligand, development

## Abstract

All living organisms must develop mechanisms to cope with and adapt to new environments. The transition of plants from aquatic to terrestrial environment provided new opportunities for them to exploit additional resources but made them vulnerable to harsh and ever-changing conditions. As such, the transmembrane receptor-like kinases (RLKs) have been extensively duplicated and expanded in land plants, increasing the number of RLKs in the advanced angiosperms, thus becoming one of the largest protein families in eukaryotes. The basic structure of the RLKs consists of a variable extracellular domain (ECD), a transmembrane domain (TM), and a conserved kinase domain (KD). Their variable ECDs can perceive various kinds of ligands that activate the conserved KD through a series of auto- and trans-phosphorylation events, allowing the KDs to keep the conserved kinase activities as a molecular switch that stabilizes their intracellular signaling cascades, possibly maintaining cellular homeostasis as their advantages in different environmental conditions. The RLK signaling mechanisms may require a coreceptor and other interactors, which ultimately leads to the control of various functions of growth and development, fertilization, and immunity. Therefore, the identification of new signaling mechanisms might offer a unique insight into the regulatory mechanism of RLKs in plant development and adaptations. Here, we give an overview update of recent advances in RLKs and their signaling mechanisms.

## 1 Introduction

The plant kingdom consists of species with a wide range of morphological diversity ranging from the simple members, such as mosses that have a thickness of the two-cell layer, to the more complex and advanced flowering plants, such as the Brassicaceae family having structures like vascular tissues, stomata, and flowers. This evolutionary journey from simple to complex, finally becoming more adaptive to the ever-changing environment, is challenging due to its sessile nature. Due to these challenges, plants have evolved conserved mechanisms that allow them to utilize different sensory proteins to recognize and perceive various environmental cues and respond appropriately to ensure optimal fitness. Cell-to-cell communication is essential to both plants and animals, as both are multicellular organisms that require this highly regulated mechanism for environmental adaptation and development. Eukaryotic protein kinases (EPKs) are a kinase superfamily that facilitates such cell-to-cell communication and intracellular signal transduction by catalyzing the transfer of ϒ-phosphate from an ATP to a hydroxyl group of serine/threonine or tyrosine residue of the polypeptide. The autophosphorylation of kinases themselves and transphosphorylation of other substrates cause a conformational change, thus activating a series of specific signaling components (Hanks and Hunter, 1995; Lehti-Shiu et al., 2012). There are over 1,000 EPKs in *Arabidopsis* (The Arabidopsis Genome, 2000), while there are only approximately 500 of them in humans (Manning et al., 2002), suggesting the importance of EPKs in regulating numerous aspects of cellular regulation in eukaryotes, particularly in plants. The first-ever evidence that suggests the process of phosphorylation in plants was found in leaf discs of Chinese cabbage (Ralph et al., 1972). Subsequently, another study based on duckweed showed that phosphorylation takes place at the serine residue in the plant ribosomes (Trewavas, 1973). In 1973, the first-ever EPK was identified and purified from pea plants; however, it was not until 1990 that the first EPK sequences were identified in the rice and pea plants (Keates, 1973; Lawton et al., 1989). Several kinase families, such as calcium-dependent protein kinases (CDPKs), NIMA-related kinases (NEKs), mitogen-activated protein kinases (MAPKs), glycogen synthase kinases (GSKs), and receptor-like kinases (RLKs) are the members of EPK family, with their unique functions and structure characteristics (Shiu and Bleecker, 2003; Lehti-Shiu and Shiu, 2012).

**Figure 1:**
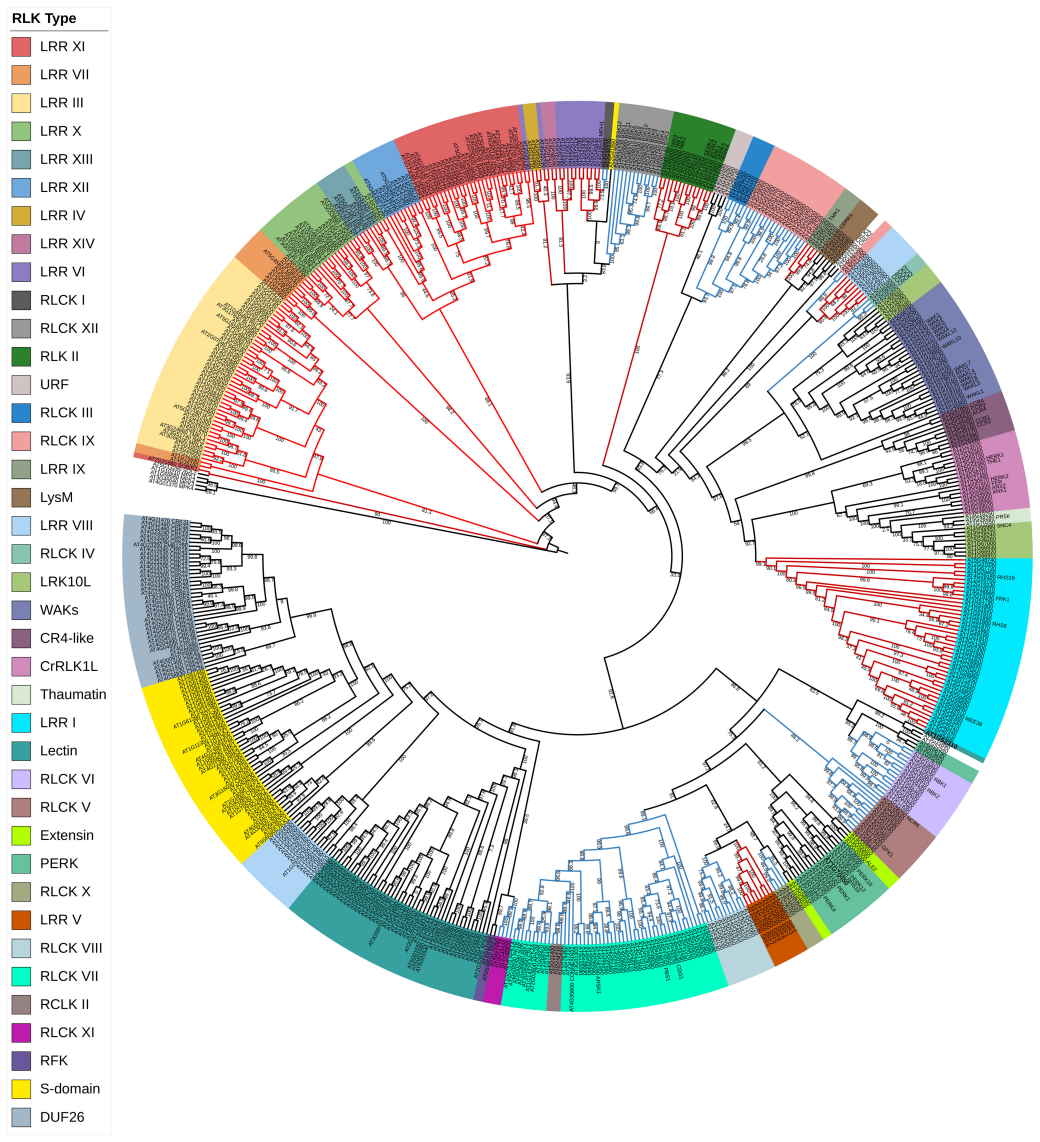
Phylogenetic classification of *Arabidopsis thaliana* RLKs. *Arabidopsis* RLK protein sequences were retrieved from the *Arabidopsis* information resource databases (https://www.arabidopsis.org/). All the retrieved sequences were aligned with MAFFT, and the tree was inferred by the maximum likelihood (ML) method with 1,000 bootstraps using IQ-TREE software (Trifinopoulos et al., 2016). Different taxon colors represent various RLK subgroups. The red color branches denote the LRR-RLK family, while the blue branches represent the RLCK family. RLKs, receptor-like kinases; LRR, leucine-rich repeat.

The two most dominant plant receptor families are cell surface-localized receptors RLKs and receptor-like proteins (RLPs) (Couto and Zipfel, 2016; Tang et al., 2017). RLKs are one of the most significant groups of plant cell surface receptors that arose from a common ancestor in green algae, playing a critical role in plant growth and development. In plants, cell-to-cell communication is regulated by their unique and distinctive structure, a hallmark of RLKs. RLK family is the largest gene family of the EPK superfamily with more than 600 members in *Arabidopsis*, which represent 2.5% of its total genome (**Figure 1**), and 1,132 members in rice (Shiu et al., 2004). The first RLK was discovered and cloned in maize, and it was found that it has a large putative N-terminal extracellular domain (ECD) together with a transmembrane domain (TM) and a catalytic intracellular kinase domain (KD) (Walker and Zhang, 1990). This discovery led to a distinctive perspective of understanding protein kinases in plants that co-opted a different class of EPKs for function in ligand-based transmembrane signal perception and transduction.

The plant RLKs have also been identified in animals, collectively called RLK/Pelle family, which forms a monophyletic clade, but no RLKs have been reported in fungi so far (Shiu and Bleecker, 2001b; Shiu and Bleecker, 2003). Pelle kinase is the only member from the RLK/Pelle family in *Drosophila*, while the human interleukin receptor-associated kinases (HsIRAK-1, HsIRAK-2, HsIRAK-M, and HsIRAK-4), responsible for adaptive and innate immunity, also belong to this family (Shelton and Wasserman, 1993; Janssens and Beyaert, 2003; Lehti-Shiu et al., 2009). The RLKs resemble the animal receptor serine/threonine kinases and receptor tyrosine kinase (RTKs) in their basic structure. The animal RTKs are distinguished from the plant RLKs by their tyrosine kinase specificity, except the transforming growth factor β (TGF-β) receptor in metazoans, which is serine/threonine kinase similar to plant RLKs (Shiu and Bleecker, 2001b; Cock et al., 2002). Some plant RLKs have been reported with both serine/threonine and tyrosine kinase specificity. For example, brassinosteroid insensitive 1 (BRI1), BRI1-associated kinase 1 (BAK1), HAESA (HAE), pollen-expressed kinase 1 (PRK1), and nod factor receptor 1 (NFR1) can phosphorylate at serine/threonine residue as well as at tyrosine residue (Oh et al., 2009; Madsen et al., 2011). In addition to that, both RTKs and RLKs undergo ubiquitination and endocytosis for system desensitization following activation (Martins et al., 2015). In addition, animal RTKs and plant RLK signaling shares similar downstream elements, such as MAPK cascades and reactive oxygen species (ROS) production (Liang and Zhou, 2018).

A large number of RLKs are found that only possess the cytoplasmic KD but lack the ECD. These members of RLKs are known as receptor-like cytoplasmic kinases (RLCKs) and play a vital role in regulating plant cellular activities in response to biotic/abiotic stresses and endogenous extracellular signaling molecules (Shiu and Bleecker, 2001a). The number of RLCKs in *Arabidopsis* and rice is 149 and 379, respectively (Shiu et al., 2004; Vij et al., 2008), while the reported number of RLCKs in maize is 162 (Fan et al., 2018b). RLPs, on the contrary, lack the KD but possess the ECD and TM or a glycosylphosphatidylinositol anchor. Hence, the RLPs are dependent on RLKs to induce transmembrane signaling (Liang and Zhou, 2018). The number of RLPs in *Arabidopsis* and rice is approximately 170 and 90, respectively (Fritz-Laylin et al., 2005; Li et al., 2016b).

In this review, we summarize the role of RLKs in crucial diverse processes in plants. Our objectives are to discuss the RLK’s evolution, crystal structure, classification, role in plant growth and development, and immunity. This review also highlights the vital role of RLCKs in RLK signaling.

## 2 Evolution and expansion of RLKs

During evolution, new species arise when countless tiny variations accumulate in the genome of an existent organism, which are heritable characteristics selected by natural selection. In short, this theory of evolution mainly emphasizes the accumulation of microevolution that is beneficial to the survival of an organism to achieve macroevolution, and eventually, new species emerge (Darwin, 1859; Reznick and Ricklefs, 2009). During this process, a gene duplicates and neofunctionalizes. However, the experimental validation of macroevolution is almost impossible due to the lack of various intermediate species between different lineages. EPKs are a monophyletic clade that controls almost all aspects of the eukaryotic life cycle (Hanks et al., 1988; Shiu and Bleecker, 2001b; Cock et al., 2002). Their sequences and structures are diverse and conserved, which are considered a good model for studying gene duplication and functional diversification (Hanks et al., 1988; Hanks and Hunter, 1995). RLKs are the most diverse class of protein kinases in plants, sensing extracellular signals and triggering the downstream responses, which play a massive role in the process of plant adaptation to the terrestrial environment (Shiu et al., 2004; Gish and Clark, 2011). Therefore, elucidating the functional evolution of plant RLKs is significant. Since all eukaryotic kinases are derived from a common ancestor, their diverse molecular and biological function is achieved through gene duplication and neofunctionalization. Gene duplication is the primary source of evolving new genes and contributes to unique biological traits and even speciation. Therefore, gene duplication and neofunctionalization can be the cornerstones of evolution (Innan and Kondrashov, 2010; Kaessmann, 2010). Most redundant gene copies after gene duplication are likely to be non-functionalized and thus are often deleted from the genome in a few million years (Lynch and Conery, 2000). However, in some cases, a few copies that acquire new functions are retained and diversified (Ohno, 1970; Force et al., 1999; Bergthorsson et al., 2007). That is why a surprising number of protein kinases have emerged during the genome expansion (The Arabidopsis Genome, 2000).

The plant RLKs are more similar to animal RTKs than other plant protein kinases in other groups, such as CDPKs, MAPKs, and GSK3s, and are within a monophyletic protein kinase group. The serine and/or threonine kinase is the ancestor of RLKs, while RTKs evolved from tyrosine kinases (Cock et al., 2002; Gish and Clark, 2011). RLKs dramatically expanded in land plants, especially angiosperms, evident of large family size in plants than its animal counterpart. To understand the evolutionary dynamics of RLKs, several initial comprehensive studies on RLKs’ identification, classification, and evolution and expansion have greatly helped gain further insight into this gene family in plants (Shiu and Bleecker, 2001a; Shiu and Bleecker, 2001b; Shiu et al., 2004; Lehti-Shiu et al., 2009; Lehti-Shiu et al., 2012). Further studies confirmed that RLKs are present in most Charophytes and all land plants, the kinase-associated RLKs first appeared in Chlorophytes, and more than half of analyzed Chlorophytes have RLKs. In 2007, the first cDNA-based report of algae containing RLKs came from *Closterium ehrenbergii* and *Nitella axillaris* with 14 and 13 RLKs, respectively, containing an ECD, TM, and a KD (Sasaki et al., 2007). *C. braunii*, another Charophyte, was initially found to have seven lysine motif kinase (LysM) RLKs in its genome (Nishiyama et al., 2018), which was later identified to have 435 RLKs in total (Dievart et al., 2020), with leucine-rich repeat (LRR), proline-rich, LysM, and malectin as their major domain families. Interestingly, the domain families such as self-incompatibility (S-domain), wall-associated kinases (WAKs), CRINKLY4-like kinase (CR4-like), and lectin (C-Lec) were absent from not only *C. braunii* but also from Zygnematophyceae and Coleochaetophyceae, suggesting their land plant specificity, as these domain families are found in basal land plants (Dievart et al., 2020; Gong and Han, 2021). Similarly, the first receptor with canonical configuration was found in the earliest Streptophyta, *Klebsormidium flaccidum*, with 94 RLKs with a TM acquisition, an ECD, and a KD. The LRR, proline-rich, extensin, and C-Lec were the only kinase-associated ECDs found in the RLKs of this genome, suggesting that the first fusion between different domains of a kinase receptor occurred in this lineage (Hori et al., 2014; Dievart et al., 2020). In many other algae members, different independent domains such as KD or conserve motifs of ECD have been found as discrete elements rather than a complete receptor, suggesting that the receptor reshaping had already been set off in green algae just before the divergence of Charophytes into the land plants (Lehti-Shiu et al., 2009). In the earliest-known diverging Charophytes, *Mesostigma viride* and *Chlorokybus atmophyticus* from Mesostigmatophyceae and Chlorokybophyceae, respectively, no RLKs were initially identified by using available expressed sequence tag (EST)/cDNA data (Simon et al., 2006; Lemieux et al., 2007). However, a recent study reported 6 and 11 RLKs in *M. viride* and *C. atmophyticus*, respectively, suggesting that kinase-associated LRR and C-Lec trace back to the earliest Streptophyte (**Table 1**) (Gong and Han, 2021).

**Table 1:** Numbers of RLKs identified in different plant species.

Classification	Species	Number of RLKs	Reference
Glaucophytes	*Cyanophora paradoxa*	7	(Dievart et al., 2020; Gong and Han, 2021)
Prasinodermophyta	*Prasinoderma coloniale*	1	(Gong and Han, 2021)
Chlorophytes	*Chlamydomonas reinhardtii*	∼4	(Lehti-Shiu et al., 2009; Gong and Han, 2021; Yan et al., 2023)
	*Chlamydomonas eustigma*	2	(Gong and Han, 2021)
	*Haematococcus pluvialis*	4	(Dievart et al., 2020)
	*Ulva mutabilis*	1	(Dievart et al., 2020)
	*Coccomyxa subellipsoidea*	2	(Dievart et al., 2020)
	*Micromonas commoda*	1	(Gong and Han, 2021)
	*Coccomyxa subellipsoidea*	2	(Gong and Han, 2021)
	*Monoraphidium neglectum*	2	(Gong and Han, 2021)
	*Raphidocelis subcapitata*	2	(Gong and Han, 2021)
	*Dunaliella salina*	2	(Gong and Han, 2021)
	*Gonium pectoral*	2	(Gong and Han, 2021)
	*Volvox carteri f. nagariensis*	∼2	(Dievart et al., 2020; Gong and Han, 2021)
Charophytes	*Klebsormidium flaccidum*	94	(Dievart et al., 2020)
	*Klebsormidium nitens*	91	(Gong and Han, 2021)
	*Chara braunii*	∼435	(Dievart et al., 2020; Gong and Han, 2021)
	*Closterium ehrenbergii*	14	(Sasaki et al., 2007)
	*Nitella axillaris*	13	(Sasaki et al., 2007)
	*Mesostigma viride*	6	(Gong and Han, 2021)
	*Chlorokybus atmophyticus*	11	(Gong and Han, 2021)
	*Mesotaenium endlicherianum*	75	(Gong and Han, 2021)
	*Spirogloea muscicola*	142	(Gong and Han, 2021)
Liverworts	*Marchantia polymorpha*	∼234	(Dievart et al., 2020; Gong and Han, 2021)
Mosses	*Physcomitrella patens*	329	(Lehti-Shiu et al., 2009)
Lycophytes	*Selaginella moellendorffii*	∼324	(Dievart et al., 2020; Gong and Han, 2021)
Gymnosperms	*Ginkgo biloba*	824	(Dievart et al., 2020)
	*Gnetum montanum*	537	(Dievart et al., 2020)
	*Pinus lambertiana*	3,548	(Dievart et al., 2020)
	*Pinus taeda*	2,286	(Dievart et al., 2020)
	*Picea abies*	775	(Dievart et al., 2020)
Angiosperms	*Amborella trichopoda*	∼424	(Dievart et al., 2020; Yan et al., 2023)
	*Panax ginseng* C.A. Meyer	563	(Lin et al., 2018)
	*Oryza sativa*	1,132	(Shiu et al., 2004)
	*Zea mays*	787	(Yan et al., 2023)
	*Triticum aestivum*	3,889	(Yan et al., 2023)
	*Brachypodium distachyon*	839	(Yan et al., 2023)
	*Aegilops tauschii*	1,183	(Yan et al., 2023)
	*Populus trichocarpa*	1,192	(Lehti-Shiu et al., 2009)
	*Arabidopsis thaliana*	610	(Shiu et al., 2004)

RLKs, receptor-like kinases.

Several recent attempts have been made to identify RLKs in Chlorophytes. *Chlamydomonas reinhardtii* was the first reported Chlorophyte to have two RLKs but none in *Ostreococcus tauri* (Lehti-Shiu et al., 2009). A recent study identified four RLKs in *C. reinhardtii* with one C-Lec, one L-Lec, and two RLCK members (Yan et al., 2023). Similarly, Gong and Han (2021) studied a total of 18 Chlorophytes, and nine were identified to have RLKs. In addition, RLKs were also present in Prasinodermophyte (*Prasinoderma coloniale*) and Glaucophyte (*Cyanophora paradoxa*) but none in Rhodophytes, giving a patchy distribution in Chlorophytes (**Table 1**) (Gong and Han, 2021). Because of this patchy distribution, the RLKs in Chlorophytes do not form a monophyletic group, which can be explained either by a single origin followed by multiple losses or by multiple horizontal gene transfers to Chlorophytes.

In non-seed land plants, moss (*Physcomitrella patens*) and lycophyte (*Selaginella moellendorffii*), the number of identified RLKs is 329 and 324, respectively, accounting for 0.36% and 0.30% of their total protein-coding genes, respectively (**Table 1**) (Lehti-Shiu et al., 2009; Dievart et al., 2020; Gong and Han, 2021). The liverwort (*Marchantia polymorpha*) represents all the major domain families except the domain of unknown function 26 (DUF26) that first appeared in *S. moellendorffii* (Vaattovaara et al., 2019; Gong and Han, 2021). In angiosperms, however, on average, there are approximately 900 RLKs in 53 monocots and 127 dicots species analyzed, which account for 0.67% to 1.39% of their total genome (Shiu et al., 2004; Zulawski et al., 2014). These ratios indicate that the RLK family continued to expand in vascular plants after divergence from the algae and then the lower plant lineage, such as moss, as there are as many as 1.9, 3.3, and 3.6 times RLK members in *Arabidopsis*, rice, and popular than that of moss, respectively. Moreover, the RLK family is greatly expanded in *Triticum aestivum* with 3,889 members, which is approximately 6.4 times those in *Arabidopsis* and 3.4 times in rice (Yan et al., 2023). All these studies suggest that the expansion of RLKs was necessary for the acquisition of their new role in plant survival and adaptation since this expansion is associated with genome complexity rather than genome size (Lehti-Shiu et al., 2009).

## 3 Conserved crystal structure of RLKs and EPKs

**Figure 2:**
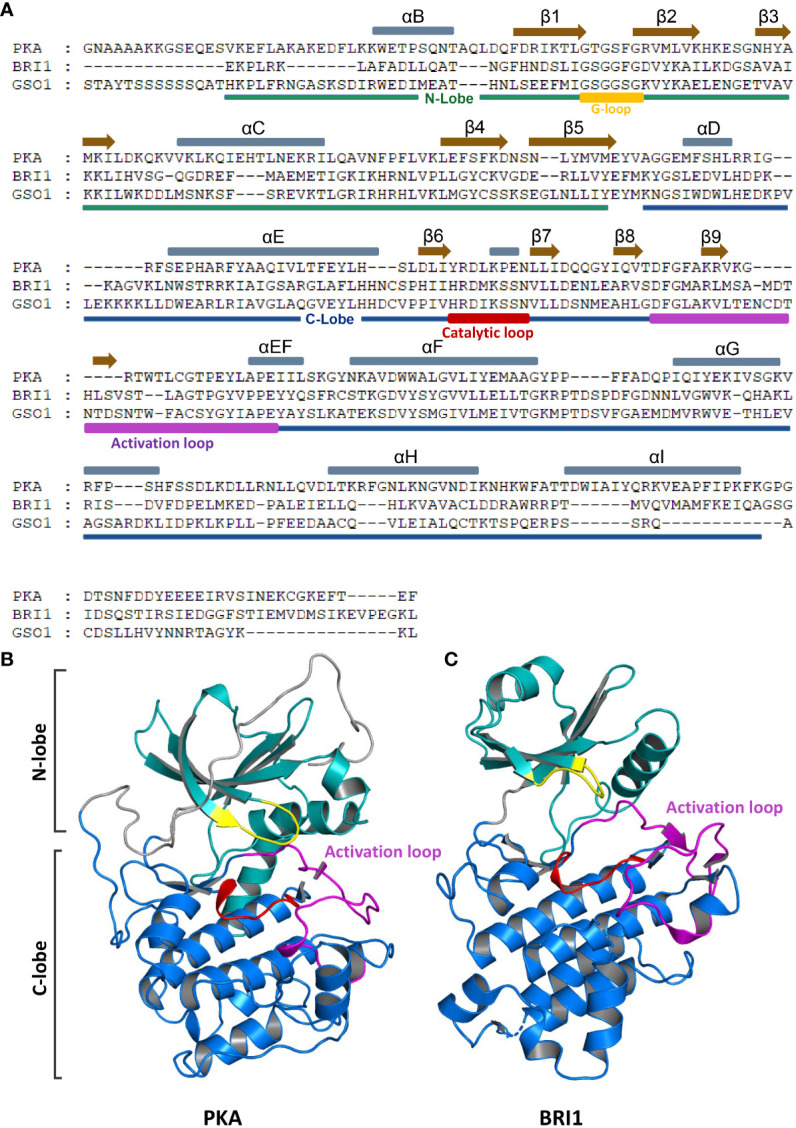
Domain organization and crystal structure of RLKs and EPKs. **(A)** N-lobe, C-lobe, αC helixes, and β-sheets of RLK (BRI1 and GSO1) and EPK (PKA) are presented in different colors. Gray and brown bars above the sequence represent αC helixes and β-sheets, respectively. The N-lobe and C-lobes are depicted below the sequences in green and blue bars, respectively. Activation loop, G-loop, and catalytic loop are marked in purple, yellow, and red, respectively. **(B, C)** Crystal structures of PKA **(B)** and BRI1 **(C)**. Different domains are represented in different colors as shown in panel **(A)**. RLKs, receptor-like kinases; EPKs, eukaryotic protein kinases.

In Eukaryotes, post-translational modification of proteins is the most widespread and conserved process carried out through the common mechanism of phosphorylation. The phosphorylation-catalyzing enzymes are regulated rigorously because phosphorylation controls the activity of a large number of proteins (Olsen and Mann, 2013). In EPKs, the primary regulatory mode is phosphorylation, together with many different ways to regulate EPKs. The cyclic-AMP protein kinase A (PKA) was first identified as a kinase where the C-subunit could phosphorylate the glycogen phosphorylase kinase (**Figure 2**) (Walsh et al., 1968). Since then, the PKA has been used as a model kinase, which opened up new insights into the concept of kinase signaling. There are many regulatory hotspots that regulate phosphorylation together, but one critical regulatory hotspot is the “activation loop”, where the phosphorylation events occur (Nolen et al., 2004; Beltrao et al., 2012; Bojar et al., 2014). The dynamic assembly of the regulatory spine is induced by the major mechanism of activation loop phosphorylation site activated through phosphorylation (Taylor et al., 2012; Meharena et al., 2013; Taylor et al., 2015). The whole activation process is based on the regulatory spine and is accompanied by structural changes.

The EPKs are defined by the conserved kinase core that consists of approximately 250 amino acid residues, essential for the activation process. Initially, the kinase core was divided into 12 subdomains based on the conserved residues in the motifs. The crystal structure analysis of EPKs revealed that these motifs could be defined based on their putative functions (Knighton et al., 1991a; Knighton et al., 1991b; Hanks and Hunter, 1995). The main kinase core consists of two lobes, a smaller N-terminal lobe (N-lobe) and a larger C-terminal lobe (C-lobe) (**Figures 2A–C**). The N-lobe is required for the ATP binding, while the substrate recognition happens at the C-lobe. In protein-activated conformation, a central network of hydrophobic amino acids (spines) connects these lobes non-covalently, resulting in independent folds (Kornev et al., 2008). Each lobe is made up of α-helixes and β-strands (subdomains), and the lobes form a deep cleft that binds adenosine triphosphate (ATP). Phosphoryl transfer occurs at the cleft’s outer edge, where the ϒ-phosphate is positioned. Through opening and closing, the active cleft mediates the catalysis, allowing the transfer of the phosphate group and the release of the nucleotide (Taylor et al., 2012).

The N-lobe consists of five-stranded β-sheets that are antiparallel in configuration involved in the ATP binding. A single conserved helix αC is located between β3 and β4. In addition to αC, there is another shorter helix, αB, which is not conserved in EPKs. A glycine-rich loop (G-loop) links the β1 to the β2, which packs on top of the ATP. In activated form, the N-terminus of αC interacts with the activation loop, while the C-terminus forms part of the active site cleft (Taylor et al., 2012; Beenstock et al., 2016). The αC serves as an allosteric hotspot, and phosphorylation of the activation loop induces the rotation of the αC helix inward to a favorable position, which enables the formation of αC-Glu and β3-Lys salt bridge and a domain encloser between the N-lobe and C-lobe (Beenstock et al., 2016; Kim et al., 2017). On the contrary, the C-lobe mostly consists of α-helixes, αD, αE, αEF, αF, αG, αH, and αI together with four β-strands, β6, β7, β8, and β9. β6 and β7 are connected by a catalytic loop that contains Asp residue, required for the activation of hydroxyl of the phosphoacceptor residue of the substrate (Beenstock et al., 2016; Kim et al., 2017). Between the β8 and β9 is a DFG motif (Asp-Phe-Gly) located right below the C helix and the G-loop between the two lobes, referred to as magnesium positioning loop because the conserved Asp residue binds to the catalytic magnesium ion (Meharena et al., 2013). The DFG motif is the start site of the activation loop, while the APE motif is the end site. The APE motif serves as an anchor for the activation loop (**Figure 2A**) (Gogl et al., 2019).

The crystal structure-based studies of RLKs allow us to understand the kinase activation mechanism and their transphosphorylation activity in plants. The RLK BRI1 crystal structure and interaction, and kinase activation mechanism with coreceptor BAK1 have been studied extensively. The BRI1 has a dual specificity of serine/threonine and tyrosine kinases (Friedrichsen et al., 2000; Oh et al., 2009). The catalytically competent activation loop becomes active due to the phosphorylation of Thr1039, Ser1042, and 1044. The protein interaction surface is located on the C-lobe of the BRI1 that heterodimerizes with somatic embryogenesis receptor-like kinase (SERK) coreceptor forming catalytically competent rearrangement. This interaction is regulated by the BRI1 inhibitor BKI1 (**Figure 2C**) (Bojar et al., 2014). For the BRI1 signaling regulation, both BAK1 and BKI1 compete for BRI1 heterodimerization and transphosphorylation (Wang et al., 2014). The island domain (ID) constituted of 70 amino acid residues is required for the BR perception during BR signaling (Li and Chory, 1997). A recent study showed that two conserved amino acids Tyr597 and Tyr 599 are required by BRI1 for BR perception. Mutating these two amino acids in the ID abolished the BR signaling by knocking out or knocking down the phosphorylation activity of BES1 (Li et al., 2022). Previous studies proved that the activation loop, αC helix, and αC-β4 loop are the most critical hotspots for kinase activation, and any abnormality in these regions can lead to the complete eradication of phosphorylation activity and cancer-related diseases in humans (Taylor et al., 2012; Gogl et al., 2019; Yeung et al., 2020). The BRI1 has been shown to allosterically utilize these hotspots together with β4–β5 located in two subdomains to specify its kinase function from GASSHO1 (GSO1). Replacing or deleting either of the subdomains will block the kinase activity in BRI1 and GSO1 (Ali et al., 2021). BAK1-interacting receptor-like kinase 2 (BIR2) is an enzymatic pseudokinase regulator of different signaling pathways that competes for BAK1 (Blaum et al., 2014; Halter et al., 2014). The crystal structure of BIR2 showed that the β1 strand blocks the ATP-binding pocket, and the amino acid variation in the conserved P-loop of kinase core in BIR2 blocks this protein from ATP coordination (Blaum et al., 2014). The structure-based molecular mechanism of GSO1/2 interaction with ligand Casparian strip integrity factors (CIFs) showed that GSO1/2 have evolved unique binding properties in their peptide to control different developmental processes through their signaling mechanism (Okuda et al., 2020). The crystal structural analysis of SOBIR1 displayed an unusually longer β3-αC loop, which together with Thr529 plays a crucial role in SOBIR1-induced *Nicotiana benthamiana* cell death response (Wei et al., 2022).

## 4 RLK classification

In *Arabidopsis* and rice, the RLKs are the largest family of the protein kinase superfamily, which has approximately 610 and 1,132 members, respectively, suggesting the importance of RLKs in plant growth and development (Shiu et al., 2004; Vij et al., 2008). However, not all RLKs are plant receptors; instead, some act as coreceptors or scaffold components of the complex, such as BAK1 and FERONIA (FER), a malectin-like receptor kinase (Li et al., 2002; Chinchilla et al., 2007; Stegmann et al., 2017). Interestingly, in *Arabidopsis*, out of 610 RLKs, 417 are RLK encoding genes, and 149 are RLCKs. The remaining numbers are RLK entities. Similarly, in rice, there are 379 RLCKs out of 1,132 RLKs (Shiu and Bleecker, 2001b; Shiu et al., 2004; Vij et al., 2008). Based on their versatile ECD, the RLKs can be divided into the following major subgroups.

The RLK classification includes LRR receptor-like kinase (LRR-RLKs), S-domain, lectin (C-Lec, L-Lec), WAKs, CR4-like, extensin, proline-rich extensin-like (PERK), LysM, thaumatin-like, DUF26 (also known as cysteine-rich receptor-like kinases; CRKs), *Catharanthus roseus* receptor-like kinase 1-like (CrRLK1L), leaf rust kinase-like (LRK), receptor-like kinase in flower (RKF), and kinase with unknown function (**Figure 1**). The last group of the RLKs has not been reported in *Arabidopsis*, while the rest are as follows.

### 4.1 LRR-RLKs

LRR-RLKs represent the largest class of RLKs and have more than 200 RLK encoding genes that are further divided into 15 subclasses (Shiu and Bleecker, 2001a). The first LRR-RLK was reported as a putative transmembrane protein kinase in *Arabidopsis*, which was later identified as transmembrane kinase 1 (TMK1) (Bleecker, 1991; Chang et al., 1992). Recently, LRR-RLKs have been identified and classified in numerous plant species. For instance, there are total of 379 LRR-type RLK identified in *Populus trichocarpa* (Zan et al., 2013), 531 in *T. aestivum* (Shumayla et al., 2016a), 119 and 67 in *P. patens* and *S. moellendorffii*, respectively (Liu et al., 2017), 1,641 LRR-RLKs in four *Gossypium* species (*Gossypium arboreum*, *Gossypium barbadense*, *Gossypium hirsutum*, and *Gossypium raimondii*) (Sun et al., 2018), 437 LRR-RLKs in *Saccharum spontaneum* (Cheng et al., 2021), and 444 LRR-RLKs in *Brassica napus* (Song et al., 2022). The KD is highly conserved among the LRR-RLKs, while ECD is variable with a different number of LRRs, which enables them to recognize a variety of hormonal or peptide-based ligands to control distinct functions (Shiu and Bleecker, 2001b; Gou et al., 2010).

### 4.2 S-domain

S-domain ECD resembles the ECD of S-locus-specific glycoprotein (SLG) in *Brassica* with a shared homology of approximately 52%, known to regulate the self-incompatibility of pollen–stigma interaction in mustard (Nasrallah et al., 1988). The S-domain class of RLKs is composed of 40 members in *Arabidopsis* that have three characteristic subdomains in their ECD: B-Lec (bulb-type lectin), which contains a highly conserved stretch of 40 residues, S-locus, and a 13 conserved cysteine-rich residue cluster called PAN (Nasrallah et al., 1988; Shiu and Bleecker, 2001a).

### 4.3 WAKs

The WAK family is the third type of RLKs described to have cysteine-containing epidermal growth factor (EGF) motifs in its ECD (Kohorn et al., 1992). WAKs are known to play a role in cell wall integrity and development to protect the plant from injuries during biotic and abiotic stresses. They do so by interacting with pectin or pectin-based molecules in response to changes in cellulose contents during a pathogen attack (Verica and He, 2002; Kohorn, 2015). WAKs have been identified and characterized in several plant species. For instance, *Oryza sativa* was found to have 125 OsWAKs (Zhang et al., 2005); *G. arboreum*, *G. raimondii*, and *G. hirsutum* genomes consist of 58, 66, and 99 WAKs, respectively (Zhang et al., 2021b); *Solanum tuberosum* L. has 29 WAKs (Yu et al., 2022); 38 WAKs were characterized in *N. benthamiana* (Zhong et al., 2023).

### 4.4 Lec-RLKs

Lec-RLK is the large class of RLKs, only second to LRR-RLKs in plants, playing various roles in plant development and stress tolerance. The Lec-RLK family has three types of lectins, G, L, and C lectin, but only L-Lec and C-Lec are known to exist in *Arabidopsis* with 47 encoding gene members (Shiu and Bleecker, 2001a; Shiu et al., 2004). They can interact with different kinds of carbohydrate molecules such as glucose-mannose, galactose-GlcNAc, and chitobiose (Sharon and Lis, 1990; Bellande et al., 2017). There is only one C-Lec in *Arabidopsis*, which is calcium-dependent for their ligand binding, which resembles the mammalian calcium-binding lec motifs (Shiu and Bleecker, 2001a; Shiu and Bleecker, 2001b; Cambi et al., 2005). In addition to *Arabidopsis*, one C-type, 22 L-type, and 23 G-type Lec-RLKs associated with phytohormones and stress responses were identified in *Cucumis sativus* L. (Lv et al., 2020). In 2021, 73 putative Lec-RLKs were identified from *Vigna radiata* L., classified into three subfamilies (Singh et al., 2021). Similarly, the peanut plant (*Arachis hypogaea*) genome constitutes 1,311 RLKs, among which 274 are Lec-RLKs classified further into three types: C, L, and G (Wang et al., 2021b). The genome-wide analysis of *T. aestivum* revealed that there are two C-lectin, 84 L-lectin, and 177 Bulb-lectin (B-lectin) in its genome (Shumayla et al., 2016b).

### 4.5 CR4-like

Maize CRINKLY4 (ACR4) was the first RLK described as crinkly-like (CR-like) RLK. It has a domain of SEVEN tandem repeats of tumor necrosis factor receptor (TNFR) that is 37 amino acids long together with another region of 26 amino acids made up of three cysteine residue tandem repeats similar to TNFR (Becraft et al., 1996; Gifford et al., 2003; Czyzewicz et al., 2016).

### 4.6 Extensin/proline-rich extensin-like kinases

Extensin is another cell wall development and integrity protein similar to WAKs that constitute Ser-(Hyp)_4_ repetition in their ECD, while the extensin-like kinases have glycosylated motifs of Ser-(Hyp)_3-5_ to maintain the integrity and structure of cell wall (Cassab, 1998; Borassi et al., 2016). The PERK1 was first characterized from *B. napus* and possesses a proline-rich domain in the ECD, similar in sequences to extensin proteins. During a pathogen attack, *PERK1* gene is induced rapidly after sensing the cell wall damage, suggesting its role in sensing cell wall modifications (Silva and Goring, 2002). Recently, the PERK family has also been identified in the *T. aestivum* L. genome with 30 genes classified into eight groups. Their involvement in plant growth processes was suggested by the *cis*-regulatory elements and expression profile at tissue developmental stages. Furthermore, it was hypothesized that several TaPERK genes were also involved in defense responses by their variable expression under biotic and abiotic stress settings (Shumayla et al., 2022).

### 4.7 LysM

The lysine motif-containing RLKs are generally known as LysM domain-containing receptor-like kinases (LYKs), which play a major role in defenses and provide fungal resistance, and are involved in chitin signaling. LYKs were identified during the quest for the Nod factor receptors during a legume rhizobial infection (Limpens et al., 2003; Madsen et al., 2003; Radutoiu et al., 2003). Recently, Yang et al. found a total of nine LysM-RLKs in *Brassica juncea* (Yang et al., 2021a). In another three *Brassica* species (*B. napus*, *Brassica rapa*, and *Brassica oleracea*), the number of the identified LysM is 17, 8, and 8, respectively (Abedi et al., 2022). Shumayla et al. identified 20 TaLysM genes that have tandem duplication with retention of function and 42 TaLysM (Motif) with neofunctionalization and pseudo-functionalization in *T. aestivum* L. (Shumayla et al., 2021). The ECD of LysM-RLKs is composed of three lysin motifs of approximately 40 amino acids long, which permits LysM-RLKs to perceive chito-oligosaccharide ligands and perform a dual role of immunity against pathogen and rhizobium–legume and mycorrhizal association in plants (Limpens et al., 2015; Buendia et al., 2018).

### 4.8 Thaumatin-like

Thaumatin-like, also known as PR5K (pathogenesis-related group 5 receptor-like kinase), is another class of RLKs involved in plant resistance against pathogen and chitinase activity. There are three members in *Arabidopsis* that also go by the name pathogen-related group 5 receptor kinases (PR5K), having 16 conserved residues of cysteine (Wang et al., 1996). In a recent study, thaumatin-like protein kinases were characterized in five cereal crops including two in *Brachypodium distachyon* and *O. sativa*, four in *Hordeum vulgare* and *Sorghum bicolor*, and 16 in *T. aestivum*. Their modulated expression in the presence of fungal pathogen, and heat, drought, and salt stress in *T. aestivum* suggested their roles in stress response (Sharma et al., 2020).

### 4.9 *C. roseus* receptor-like kinase 1-like

The first RLK of this class was identified in a higher plant, *C. roseus* (Schulze-Muth et al., 1996). *Arabidopsis* possesses 17 members of this family, while *T. aestivum* was recently identified to encode 15 CrRLK1L family genes that have 43 paralogous copies with three homeologs each, except for paralog -2-D and -7-A, which were absent (Gawande and Sankaranarayanan, 2023). Another study showed that three of 16 *O. sativa* genes are tandem-duplicated, suggesting possible functional redundancy within this family. However, integrated diurnal expression showed a functional divergence between two of these three genes (Nguyen et al., 2015). Ma et al. (2023) identified 24 CrRLK1L members in *Solanum lycopersicum* having bacterial and pathogen-associated molecular pattern (PAMP) responses during expression profile (Ma et al., 2023). The ECD of CrRLK1L comprises 450 amino acid residues that possess the carbohydrate-binding domain (malectin-like domain) (Nguyen et al., 2015; Moussu et al., 2018). The members of this class form complexes with other receptors or coreceptors or factors such as glycosylphosphatidylinositol-anchored protein to control plant development or other cell wall sensing responses (Nissen et al., 2016; Ge et al., 2019).

### 4.10 Leaf rust kinase-like

The first canonical receptor of leaf rust kinase-like (LRK10-like) was receptor-like kinase in flower 3 (RKF3) that has an ECD, a KD, and a TM but lacks distinct motifs in their ECD for signaling peptide (Takahashi et al., 1998). The LRK10 is RLK from wheat leaf rust kinase (WLRK) that is homologous to RLK10-like in sequence and has 14 conserved cysteines in a specific repeated manner of three conserved regions followed by three variable regions in the ECD (Feuillet et al., 1998; Feuillet et al., 2003; Lim et al., 2015). This unique architecture of ECD allows these RLKs to perceive different kinds of ligands, thus playing a diverse role in plant development.

### 4.11 DUF26

With 44 members in *Arabidopsis*, DUF26 (CRK) is another cysteine-rich repeat domain kinase receptor that shares similarities with lectins but is different than the S-locus glycoprotein and contains C-X8-C-X2-C motifs (Zeiner et al., 2023). These RLKs containing cysteine-rich repeats were termed cysteine-rich domain-containing receptor-like kinases (CRR RLKs), which play a role in defense and stress responses (Chen, 2001; Vaattovaara et al., 2019). Other cereal crops such as *B. distachyon*, *H. vulgare*, *O. sativa*, *S. bicolor*, and *T. aestivum* have also been reported to have 43, 37, 36, 38, and 170 CRKs respectively (Shumayla et al., 2019).

### 4.12 Receptor-like kinase in flower

The first RLK of this class was found to have an LRR malectin domain in which the LRRs are located upstream of the ECD (Takahashi et al., 1998). The RFK1 receptor has 13 LRRs, and the malectin domain is located between the TM and LRR (Dufayard et al., 2017).

## 5 Diverse roles of RLKs

The extensive expansion of RLKs in *Arabidopsis*, rice, and wheat genomes may suggest their role in the perception of a broad range of internal and external signals and stimuli. Although most of the RLK functions have not been identified, still, a large number of RLKs have been studied extensively. Based on their functions, the plant RLKs are divided into two broad categories. The first RLK category is responsible for growth and development, and the second controls plant–microbe interaction, immunity, and stress responses (Shiu and Bleecker, 2001a).

### 5.1 RLKs involved in plant growth and development

**Figure 3:**
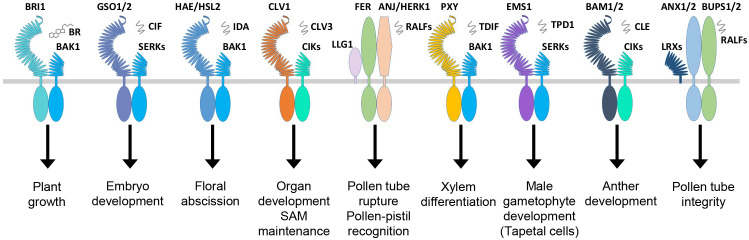
RLKs involved in plant growth and development. The plant hormone BR binds BRI1, and signaling peptides are perceived by the other membrane-localized RLKs. In *Arabidopsis*, BRI1, GSO1, HAE/HSL, CLV1, FER, PXY, EMS1, BAM1/2, and others make complexes with coreceptors and other scaffold molecules to regulate a variety of growth and developmental processes. RLKs, receptor-like kinases.

Normal growth and development are defined by the coordinated cell division, differentiation, and morphogenesis of the plant tissues controlled by a complex regulatory mechanism consisting of multiple regulatory genes working together. Several RLKs are known to regulate the development of shoot apical meristem (SAM), which develops into aerial organs in higher plants. The CLAVATA3 (CLV3) is a peptide ligand that forms complexes with CLAVATA1 (CLV1) and CLAVATA2 (CLV2, a receptor-like protein); together, they control the balance between cell differentiation and proliferation (**Figure 3**; **Table 2**) (Clark et al., 1997; Fletcher et al., 1999; Jeong et al., 1999). In *Arabidopsis*, the stem cells in the shoot meristem are regulated by the WUSCHEL (WUS), and SHOOTMERISTEMLESS (STM) interacts with the *CLV3* promoter, which enhances the WUS-mediated stem cell activity (Su et al., 2020). Recently, a study reported that CLV1 participates in auxin-dependent meristem maintenance in cool environments. The auxin-dependent floral primordium development requires CLV3 signaling, and both inflorescence fasciation and heat-induced auxin biosynthesis partially mask this role (John et al., 2023). The plant organ shape and inflorescence architecture are monitored by ERECTA (ER), which perceives the epidermal patterning factor (EPF)-like protein (EPFL) as a ligand and expressed in root and shoot apical meristem as well as developing organs (Yokoyama et al., 1998; Herrmann and Torii, 2021). The dwarf phenotype and aberrant flower development of the ER mutants suggest that this family is involved in SAM and flower development, cell proliferation, organ growth, and stomata formation (**Table 2**) (Shpak et al., 2003; Herrmann and Torii, 2021). Recently, the EPFL family member, EPFL2, has also been reported to play a role in embryogenesis. Its loss-of-function mutants displayed reduced growth in cotyledon (Fujihara et al., 2021). In *Arabidopsis*, the number of seeds and the growth of pistils and fruits are regulated through coordinated ovule patterning induced by the interaction of EPFL2 and EPFL9 with the ER and ER-like (ERL1/2) receptors (Kawamoto et al., 2020). The ER signaling pathways result from forming complexes with signaling proteins, such as SERK coreceptors and too many mouths (TMM) transmembrane proteins (Lee et al., 2012; Meng et al., 2015). Similarly, another RLK, meristematic receptor-like kinase (MRLK) expressed in root and shoot apical meristem, regulates the floral transition by interacting with the transcription factor AGL24 of the MADS-box family (Fujita et al., 2003). Several RLKs play an equally essential role in root development as the one responsible for the shoot development. One such RLK is CRINKLY4 (ACR4), which is responsible for the normal development of root apical meristem (De Smet et al., 2008; Stahl et al., 2009). ACR4 forms a complex with CLV1 in response to CLE40 peptide association, which regulates the lateral root formation and stem cell differentiation of columella (Stahl et al., 2013). The ACR4 also interacts with Protein Phosphatase 2A (PP2A) to regulate the distal root meristem development. PP2A plays a role in the ACR4 accumulation and plasma membrane localization (Yue et al., 2016). It is also known that the ACR4 regulates the homeostasis of the columella stem cells in the root by forming complexes with CLAVATA3 INSENSITIVE RECEPTOR KINASEs (CIKs) induced by CLV3/EMBRYO SURROUNDING REGION (ESR) 40 (CLE40) (Zhu et al., 2021). During epidermal cell differentiation in leaves, the ACR4 together with ABNORMAL LEAF SHAPE 2 (ALE2) positively regulates protoderm-specific gene expression (Tanaka et al., 2007). The rice OsCR4 is essential for the development of mesophyll tissues and the differentiation of vascular bundles and the epidermis by establishing a local auxin gradient to restrict OsWOX3A (Wang et al., 2020b). The member of the cysteine-rich RLK family, CRK28, is also involved in normal root morphogenesis, and its overexpression phenotype exhibits delayed and reduced lateral root growth, while the *crk28* mutant is reported to have longer and branched root (Pelagio-Flores et al., 2019). Two independent studies showed rapid root growth and navigation are regulated through the auxin-induced interaction of TMK1 with H^+^-ATPas inducing apoplastic acidification and cell expansion (Li et al., 2021b; Lin et al., 2021). S-domain RLK, *Arabidopsis* receptor kinase2 (ARK2) has been identified to interact with U box/armadillo repeat-containing E3 ligase9 (AtPUB9) mediating lateral root development under phosphate starvation (Deb et al., 2014).

**Table 2:** Major RLKs involved in plant growth and development.

Species	Gene name	Gene ID	RLK type	Function	Reference
*Arabidopsis*	*BRI1*	AT4G39400	LRR	BR-dependent growth and development	(Li and Chory, 1997; Jiang et al., 2013)
	*EMS1*	AT5G07280	LRR	Anther development, stamen elongation	(Canales et al., 2002; Zhao et al., 2002; Chen et al., 2019; Zheng et al., 2019; Bai et al., 2023)
	*BAM1/2*	AT5G65700 AT3G49670	LRR	Archesporial cell division and differentiation	(Hord et al., 2006)
	*GSO1/2*	AT4G20140 AT5G44700	LRR	Embryonic cuticle development, root growth	(Tsuwamoto et al., 2008; Pfister et al., 2014; Racolta et al., 2014)
	*CLAVATA1*	AT1G75820	LRR	Floral meristem size, shoot meristems	(Clark et al., 1997; Fletcher et al., 1999; Han et al., 2020; Shang et al., 2021)
	*HAESA/HSL*	AT4G28490 AT1G28440 AT5G65710	LRR	Floral organ abscission	(Jinn et al., 2000; Stenvik et al., 2008; Meng et al., 2016; Santiago et al., 2016)
	*ZAR1*	AT2G01210	LRR	Zygotic division	(Yu et al., 2016)
	*ER/ERL1/2*	AT2G26330 AT5G62230 AT5G07180	LRR	Root and shoot apical meristem, ovule, and seed density	(Yokoyama et al., 1998; Shpak et al., 2003; Kawamoto et al., 2020; Herrmann and Torii, 2021)
	*ALE2*	AT2G20300	Extensin	Integument development, embryo sac development, epidermal development	(Tanaka et al., 2007)
	*ACR4*	AT3G59420	CR4-like	Root apical meristem, sepal development, integument development, epidermal development	(Gifford et al., 2003; Tanaka et al., 2007; De Smet et al., 2008; Stahl et al., 2009)
	*FER*	AT3G51550	CrRLK1L	Pollen tube and root hair development	(Huck et al., 2003; Escobar-Restrepo et al., 2007; Duan et al., 2010)
	*ANX1/2*	AT3G04690 AT5G28680	CrRLK1L	Pollen tube integrity	(Boisson-Dernier et al., 2015; Liao et al., 2016; Ge et al., 2017; Du et al., 2018)
	*SERK1-4*	AT1G71830 AT1G34210 AT4G33430 AT2G13790	LRR	Plant growth and development, cell differentiation and proliferation, stomatal patterning, and floral organ abscission	(Ma et al., 2016; Li et al., 2019b; Liu et al., 2020a)
	*ARK2*	AT1G65800	S-domain	Organ maturation lateral Root, development under phosphate starvation	(Deb et al., 2014)
*Oryza sativa* (rice)	*OsBRI1*	Os01g0718300	LRR	Internode elongation, bending of the lamina joint, cell division, and elongation	(Yamamuro et al., 2000; Nakamura et al., 2006)
	*OsSERK1/2*	Os08g0174700 Os04g0457800	LRR	Enhances grain yield, leaf growth, and development	(Li et al., 2009a; Park et al., 2011; Zuo et al., 2014)
	*OsLecRK5*	Os02g0459600	Lectin	Pollen development	(Wang et al., 2020a)
	*OsLSK1*	Os01g0669100	S-domain	Improves panicle architecture and grain yield	(Zou et al., 2015)
	*OsSRK1*	Os06g0241100	S-Domain	Regulation of leaf width and salt tolerance	(Jinjun et al., 2020)
	*OsESG1*	Os01g0223800	S-domain	Regulation of early crown root development, drought resistance	(Pan et al., 2020)
	*OsRPK1*	Os05g0486100	LRR	Negative regulation of polar auxin transport, root development	(Zou et al., 2014)
*Triticum aestivum* L. (Wheat)	*TaBRI1*	TraesCS5D02G181500	LRR	Early flowering and seed yield enhancement	(Singh et al., 2016; Rafeie et al., 2020; Sharma and Khurana, 2022)

RLKs, receptor-like kinases; LRR, leucine-rich repeat.

HAE is another receptor kinase that expresses in a tissue-specific manner in the areas of abscission zones of floral organs, leaf petiole, and pedicel bases. *HAE* antisense RNA expression shows delayed abscission in floral organs, suggesting its role in the regulation of the abscission process in floral organs such as stamens, sepals, and petals (Jinn et al., 2000). HAE and HAESA-Like (HSL) interact and bind ligand peptides, inflorescence deficient in abscission (IDA) and IDL, respectively, which leads to the phosphorylation-based activation of MAP kinase signaling. The IDA ligand-based interaction of coreceptor SERK1 with HAE leads to the floral abscission pathway initiating the cell wall hydrolysis at the base of abscission organs (**Figure 3**; **Table 2**) (Santiago et al., 2016; Taylor et al., 2016).

In angiosperms, the development of male and female reproductive organs is precisely coordinated to accomplish successful fertilization. There are a number of RLKs involved in the regulation of anther and ovule development. The excess microsporocytes1 (EMS1), receptor-like protein kinases (RPK), barely any meristem (BAM1/2), etc., are known to regulate the pattering and differentiation of cell layers during anther development (Lee et al., 1996; Zhao et al., 2002; Hord et al., 2006). The EMS1 is required for the formation of tapetum during the pollen development inside the anther. Using the pan-brassinosteroid signaling, EMS1 binds the Tapetum Determinant 1 (TPD1) together with coreceptor SERK1/2 to promote the formation of tapetal precursor cells by periclinal division of parietal cells (Jia et al., 2008; Feng and Dickinson, 2010; Zheng et al., 2019; Zheng et al., 2022a). The *ems1* and *tpd1* mutants have no characteristic tapetum in anthers but produce more microsporocytes than the wild type (Canales et al., 2002; Yang et al., 2003). In another similar study, the EMS1 and BRI1 coordinate stamen elongation in *Arabidopsis* through the transcription factor BES1/BZR1 (Bai et al., 2023). The RPK2, on the contrary, regulates the anther development through differentiation of the middle layer and tapetum. RPK2 modulates the enzymes involved in lignin biosynthesis and cell wall metabolism by triggering the degradation of tapetum to control their cell fate (Mizuno et al., 2007). BAM1/2, together with RPK2, is required for early anther cell specification. A study of BAM1 and BAM2 mutant *bam1bam2* confirmed the lack of endothecium, middle layer, and tapetum in the somatic cell layers, suggesting that both of these genes are responsible for the early-stage regulation of anther differentiation (**Table 2**) (Hord et al., 2006). In *Arabidopsis*, coreceptor proteins, CIKs, interact with RPK2 and BAM1/2 to regulate early anther development. As coreceptors of BAM1/2 and RPK2, CIKs control the archesporial cell division and determine the specification of anther parietal cells (Cui et al., 2018). In rice, normal exine development is crucial for pollen grain protection and normal pollination. OsLecRK-S.7 was identified in rice as a vital regulator of pollen development. The kinase of E560K or K418E was found to be vital for the phosphorylation of OsLecRK-S.7 (Peng et al., 2020). Another lectin-type RLK, OsLecRK5, was implicated in anther development by phosphorylating UGP1, enhancing its activity in callose biosynthesis (Wang et al., 2020a). The rice OsERL is a homolog of the *Arabidopsis* ER gene family implicated in anther lobe development. Mutant *erl* displayed severe anther development defects and male sterility (Liu et al., 2021). Overexpression of a truncated version of OsLSK1 (including the extracellular and transmembrane domain of OsLSK1 without the intracellular kinase domain) increased plant height and improved yield-related components, including primary branches per panicle and grains per primary branch (Zou et al., 2015).

In plants, after the gamete formation, successful fertilization and reproduction are essential for progeny propagation. It is achieved through a productive pollen and pistil interaction guided by receptors that perceive the ovule-emitted signals in pollen tubes. The RLKs such as PRKs, LURE receptors like Male Discoverer 1 (MDIS1), and MDIS1-interacting receptor-like kinase 1 and 2 (MIK1 and MIK2) sense the ovule secreted peptide, LURE1 (Takeuchi and Higashiyama, 2012; Takeuchi and Higashiyama, 2016; Wang et al., 2016). In *Arabidopsis*, PERK5 and PERK12, which were recently identified, are necessary for cell wall assembly and ROS homeostasis, guiding proper pollen tube growth. *perk5-1* and *perk12-1* displayed male gametophytic defect and cell wall of pollen tubes with excessive accumulation of pectins and cellulose (Borassi et al., 2021). Another RLK, FER, expressed in synergids of female gametophyte, comes into play by rupturing the pollen tubes to release the sperm cells (Escobar-Restrepo et al., 2007). The *fer* mutants display the loss of rupturing ability and overgrowth of pollen tubes (Huck et al., 2003). In rice, CrRLK1L, *O. sativa* FERONIA-like receptor1 (OsFLR1, also known as DRUS1) and OsFLR2 (DRUS2) are required for maintaining architecture, reproduction, and seed yield (Li et al., 2016a; Pu et al., 2017). Moreover, apple *Malus domestica* FERONIA-like1 (MdFERL6) and tomato *S. lycopersicum* FERONIA-like (SlFERL) have been reported in fruit ripening (Jia et al., 2017; Ji et al., 2020). The consistent expression analysis of SlCrRLK1L20 with SlFERL in tomato showed that it might be involved in fruit ripening process similar to SlFERL (Ma et al., 2023). Similarly, the rice-ruptured pollen tube (RUPO) is essential for pollen tube growth and integrity (Liu et al., 2016). The pears (*Pyrus bretschneideri*), PbrCrRLK1L3, and PbrCrRLK1L26 are also known to regulate the pollen tube rupture process and growth (Kou et al., 2017). The tapetum gene expression and pollen development are regulated by a novel LRR-type PXY-like1 (PXL1) receptor perceiving CLAVATA3/EMBRYO SURROUNDING REGION-RELATED 19 (CLE19) as a ligand (Yu et al., 2023).

Normal zygotic development into an embryo is crucial for plant growth. Several studies have revealed that there are multiple signaling cascades required for embryogenesis. Zygotic arrest 1 (ZAR1) is an RLK that is expressed in the embryo sac starting from the eight-nucleated stage to the mature embryo stage. ZAR1 activates the G protein and calcium-protein signaling, thus regulating the asymmetric division of the zygote and determining the cell fate of daughter cells (Yu et al., 2016). Similarly, GSO1 and GSO2 bind the ligands CIF1 and CIF2 and play an essential role in the development of the normal epidermal surface of the embryo (**Figure 3**; **Table 2**) (Nakayama et al., 2017; Okuda et al., 2020). *gso1gso2* mutant plants show abnormalities in embryo bending and cotyledon adhesion with highly permeable epidermal structures (Tsuwamoto et al., 2008). A study reported that GSO1 specifies its function from BRI1 by merely two subdomains (Ali et al., 2021).

The development of vascular tissues is a multistep process and requires various RLKs to supervise and regulate the vascular meristem differentiation of the xylem and phloem. For example, the procambial cell division is regulated by phloem intercalated with xylem (PXY), which interacts with the ligand tracheary element differentiation factor (TDIF) to activate the WUSCHEL-related homeobox 4 (WOX4) signaling during vascular development, thus maintaining the cell polarity (**Figure 3**) (Fisher and Turner, 2007; Hirakawa et al., 2008). The BR receptors BRI1-LIKE 1 (BRL1) and BRI1-LIKE 3 (BRL3) play a role in vascular differentiation (Cano-Delgado, 2004; Zhou et al., 2004; Lozano-Elena and Caño-Delgado, 2019). BRI1-LIKE 2 (BRL2), previously known as vascular highway 1 (VH1), causes abnormal phloem transport in loss-of-function mutants (Clay and Nelson, 2002).

Phytohormone-dependent growth and abiotic stress-related regulations are vital in plant development. Hormone-based RLKs such as BRI1 and receptor dead kinase 1 (RDK1) directly or indirectly perceive the hormone to control various aspects of plant development. BRI1 directly binds to BRs, which activates the downstream signaling cascade, thus activating numerous BR-dependent regulatory genes (Li and Chory, 1997). In this process, upon BR perception, the coreceptor BAK1 associates with the BRI1 receptor, which leads to the transphosphorylation events, thus activating other downstream components of BR signaling (**Figure 3**) (Li et al., 2002). BRs are also perceived by two other receptors, BRL1 and BRL3, which are involved in the vascular differentiation in *Arabidopsis* (Cano-Delgado, 2004). A recent study found the BR activity in *Ceratopteris richardii* for the previously known non-BR receptor, BRL2 (Zheng et al., 2022b). OsBRI1 and its homologs OsBRL1 and OsBRL3 have also been reported in shoot cell elongation and cell division as well as root development (Yamamuro et al., 2000; Nakamura et al., 2006). The wheat TaBRI1 is BR-dependent and responsible for earlier flowering time and higher seed yield in *Arabidopsis* (**Table 2**) (Singh et al., 2016; Rafeie et al., 2020; Sharma and Khurana, 2022). Abscisic acid (ABA), however, is mostly reported to be involved in the regulation of abiotic stresses via indirect interactions with RLKs, unlike BRs. For instance, RDK1 interacts indirectly with ABA by recruiting a protein phosphatase, abscisic acid insensitive 1 (ABI1), on the plasma membrane regulating plant responses to abiotic stresses (Kumar et al., 2017). Similarly, the cysteine-rich receptor-like kinase CRK28 and PERK4 are also known to be the indirect regulators of ABA signaling (Bai et al., 2009; Pelagio-Flores et al., 2019). The rice S-receptor protein kinases (OsSRKa) induced by ABA, salt, and polyethylene glycol are vital for leaf width by promoting cell division in the leaf primordium and salt tolerance (Jinjun et al., 2020). Auxin-dependent root growth has been reported in *Arabidopsis* and rice. In the RHO GTPase (RAC/ROP) signaling pathway, FER as an upstream regulator controls ROS-mediated root hair development (Duan et al., 2010). The auxin- or ABA-induced OsRPK1 is a Ca(2^+^)-independent Ser/Thr kinase, negatively regulating the adventitious and lateral roots and root apical meristem. Increased plant height, tiller numbers, and growth of transgenic rice plants were reported in the OsRPK1 knockdown mutant (Zou et al., 2014). OsESG1 is an S-domain RLK that regulates early crown root development and drought resistance in rice by utilizing auxin signaling and polar auxin transport (**Table 2**) (Pan et al., 2020).

### 5.2 RLKs involved in plant defense, immunity, and stress responses

A wide range of RLKs have been identified to provide immunity to the plant against bacterial or fungal pathogens or protein elements of microbial origin. The plants sense these pathogens in the form of host-derived elicitors such as PAMPs, microbe-associated molecular patterns (MAMPs), herbivore-associated molecular patterns (HAMPs), and damage-associated molecular patterns (DAMPs). The responses resulting from these host-derived elicitors are called pathogen-triggered immunity (PTI), while the RLKs involved in this process are known as pattern recognition receptors (PRRs). For instance, the effector-triggered immunity RLKs include tomato Pto and Pto-interacting 1 (Pti1) (Martin et al., 1993; Zhou et al., 1995), *Arabidopsis* flagellin Insensitive 2 (FLS2), LRK10 (Feuillet et al., 1998), Xa21 in rice (Song et al., 1995), and PBS1 in *Arabidopsis* (Swiderski and Innes, 2001). Pti1, Pto, and PBS1 are implicated in resistance against bacterial infections. Pti1 itself does not provide immunity against bacteria; instead, it interacts with Pto to confer disease resistance (Zhou et al., 1995). Conversely, *Pto* gene in tomato confers resistance to races of *Pseudomonas syringae* pv. *tomato*, which carries the avirulence gene *avrPto* (Martin et al., 1993). Similarly, the PBS1 also mediates protection against *P. syringae* pv. *phaseolicola* (Swiderski and Innes, 2001).

**Figure 4:**
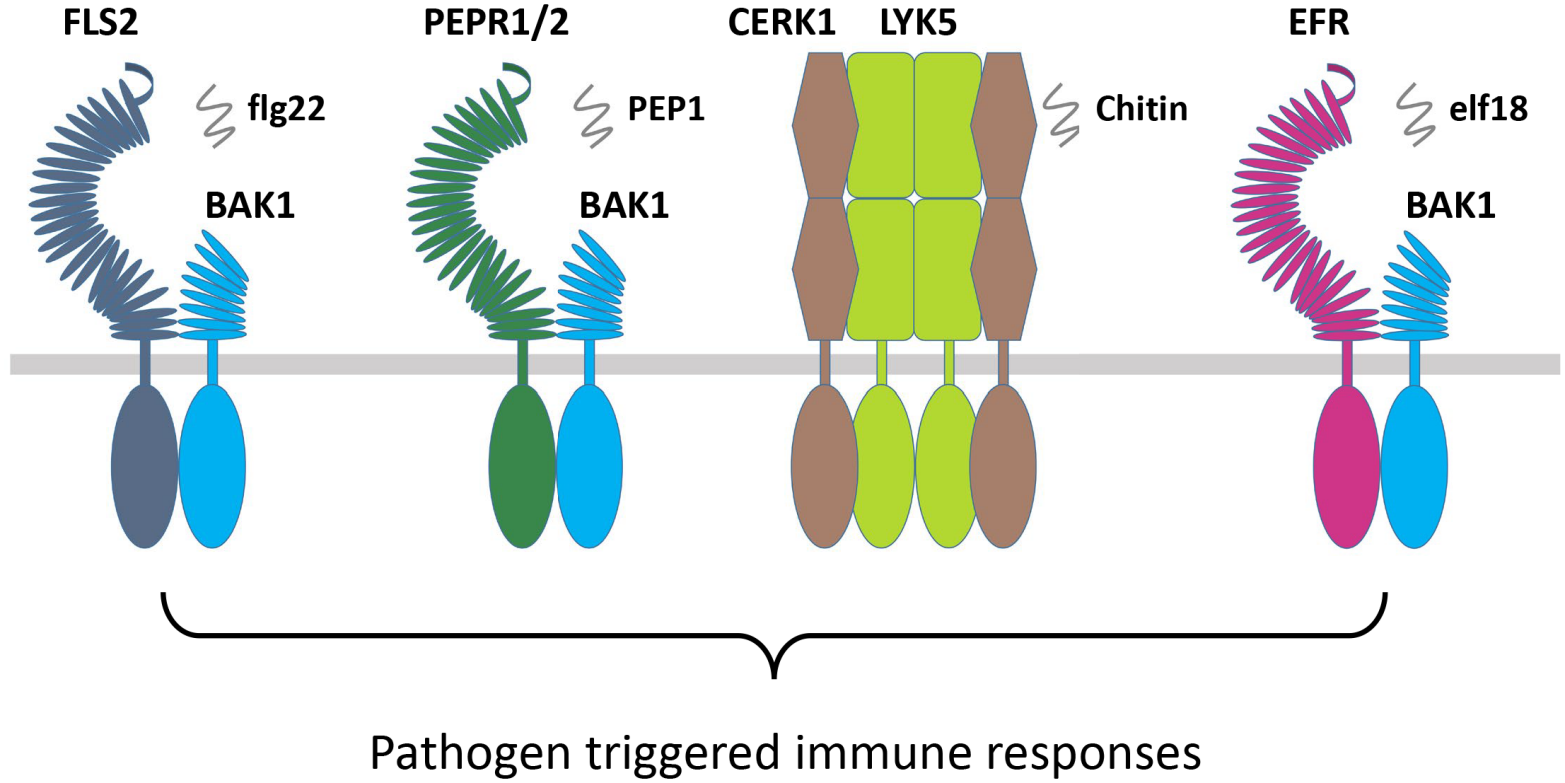
RLKs involved in pathogen immune responses. Pathogen or pathogen elicitor-based RLKs FLS2, PEPR1/2, CERK1/LYK5, and EFR interact with their coreceptors BAK1/SERKs and chitin to induce PTI responses. RLKs, receptor-like kinases; PTI, pathogen-triggered immunity.

The FLS2 and Xa21 are closely related in their sequences and belong to the same subgroup of RLKs. The FLS2 recognizes the bacterial epitope flagellin (flg22), which is bacterial elicitor PAMP, and recruits the coreceptor BAK1 or BAK1-like kinase 1 (BKK1), which subsequently activates their transphosphorylation (**Figure 4**; **Table 3**) (Gómez-Gómez et al., 1999; Gómez-Gómez and Boller, 2000). This activation leads to the phosphorylation of botrytis-induced kinase 1 (BIK1) and causes its dissociation from the FLS2-BAK1/BKK1 complex. Finally, the PTI responses are regulated through the calcium and ROS as well as the activation of MAPKs or CDPKs (Segonzac and Zipfel, 2011). The FLS2 and BAK1 interaction is negatively regulated by a pseudokinase, BIR2, which competes for the BAK1 interaction (Blaum et al., 2014; Halter et al., 2014). However, the rice *OsXa21* provides resistance against *Xanthomonas oryzae* pv. *oryzae*, which is a blight pathogen (Song et al., 1995). OsSERK2 associates with OsXa21, OsXa3, and OsFLS2 to positively regulate immune responses in rice (Chen et al., 2014). However, OsSERK1 is involved in rice growth regulation but not reported in immune responses (Zuo et al., 2014). The wheat *TaXa21* is highly homologous to *OsXa21*, cloned from wheat cultivar Xiaoyan 6 (XY 6), and involved in temperature-dependent resistance to strip rust in wheat seedlings. *TaXa21* interacts with TaWRKY76 and TaWRKY62 working as a positive regulator under high temperatures, thus providing resistance against *Puccinia striiformis* (Wang et al., 2019).

**Table 3:** Major RLKs involved in plant immunity and stress responses.

Species	Gene name	Gene code	RLK type	Function	Reference
*Arabidopsis*	*CERK1*	AT3G21630	LysM	Perception of MAMPs	(Willmann et al., 2011; Cao et al., 2014)
	*CRK2*	AT1G70520	S-domain	Pathogen response, ROS production, Ca^2+^ influx, MAPK cascade activity, callose deposition	(Hunter et al., 2019; Kimura et al., 2020)
	*CRK5*	AT4G23130	S-domain	Pathogen response, MAPK cascade activity, callose deposition	(Chen et al., 2004; Burdiak et al., 2015; Zhao et al., 2022)
	*CRK13*	AT4G23210	S-domain	Immunity against *Pseudomonas syringae*	(Acharya et al., 2007; Yadeta et al., 2016)
	*CRK22*	AT4G23300	S-domain	Pathogen response, MAPK cascade activity, callose deposition	(Yadeta et al., 2016; Zhao et al., 2022)
	*CRK28*	AT4G21400	S-domain	Resistance against *P. syringae* and induces cell death in *Nicotiana benthamiana*	(Yadeta et al., 2016)
	BAK1 (SERK3)	AT4G33430	LRR	Form complexes with RLKs to promote plant resistance against pathogens	(Chinchilla et al., 2007; Schwessinger et al., 2011; Sun et al., 2013b; Halter et al., 2014)
	*NIK1*	AT5G16000	LRR	Positively regulates plant antiviral immunity, negatively regulates plant antibacterial immunity	(Machado et al., 2015; Li et al., 2019a)
	*CDG1*	AT3G26940	RLCK	Pattern-triggered immunity and effector-triggered susceptibility in plants	(Yang et al., 2021b)
	*PEPR1/2*	AT1G73080 AT1G17750	LRR	PAMP-triggered immune responses	(Huffaker et al., 2006; Yamaguchi et al., 2006; Liu et al., 2013)
	*EFR*	AT5G20480	LRR	Involved in plant defense	(Zipfel et al., 2006; Li et al., 2009b; Schwessinger et al., 2011)
	*FLS2*	AT5G46330	LRR	Flagellin perception, innate immune response	(Gómez-Gómez and Boller, 2000; Gómez-Gómez et al., 2001; Chinchilla et al., 2007)
	*SOBIR1*	AT2G31880	LRR	Constitutive immunity, cell death positive regulator	(Gao et al., 2009; Van der Burgh et al., 2019; Wei et al., 2022)
	*PR5K*	AT5G38280	Thaumatin	Self-incompatibility and disease resistance	(Wang et al., 1996)
	*PERK1*	AT3G24550	PERK	Respond to a wound and/or pathogen stimulus	(Silva and Goring, 2002)
	*PERK4*	AT2G18470	PERK	Functions at an early stage of ABA signaling to inhibit root cell elongation	(Bai et al., 2009)
	*RLK7*	AT1G09970	LRR	Involved in the control of germination speed and the tolerance to oxidant stress	(Pitorre et al., 2010)
	*HSL3*	AT5G25930	LRR	Regulates stomatal closure and drought stress response	(Liu et al., 2020b)
*Oryza sativa* (Rice)	*OsCERK1*	Os09g0511000	LysM	Regulation of chitin-triggered immunity and arbuscular mycorrhizal symbiosis	(Shimizu et al., 2010)
	*OsXa21*	Os11g0559200	LRR	Resistance against *Xanthomonas oryzae* pv*. oryzae*	(Song et al., 1995)
	*OsSOBIR1*	Os06g0288100	LRR	Regulation of PTI response and antiviral defense	(Zhang et al., 2021a)
	*OsSERK2*	Os04g0457800	LRR	Xa21-associated immune responses	(Chen et al., 2014)
	*Pi65*	Os11g0694600	LRR	Rice blast resistance	(Wang et al., 2022)
	*OsCRK6*	Os07g35690	S-domain	NH1-mediated immunity in rice	(Chern et al., 2016)
	*OsCRK10*	Os07g35700	S-domain	NH1-mediated immunity in rice	(Chern et al., 2016)
	*SDS2*	Os01g0783800	S-domain	Positively regulation of programmed cell death and immunity, enhanced resistance to *Magnaporthe oryzae*	(Fan et al., 2018a)
	*OsSIT1*	Os02g0640500	Lectin	Mediation of salt sensitivity, regulation of ethylene homeostasis	(Li et al., 2014a)
	OsWAK11	Os02g0111600	WAKL	Regulates resistance to aluminum and copper, cell elongation	(Hu et al., 2014; Yue et al., 2022)
	*OsSTLK*	Os05g0305900	LRR	Positive regulator of salt stress tolerance	(Lin et al., 2020)
*Triticum aestivum* L. (Wheat)	*TaXa21*	TraesCS4D02G352700	LRR	Positive regulator of wheat high-temperature seedling plant (HTSP) resistance to *Puccinia striiformis* f. sp. *tritici*	(Wang et al., 2019)
	*TaCRK10*	TraesCSU02G182500	S-domain	Serves as an important sensor of *P. striiformis* infection and high temperatures	(Wang et al., 2021a)
	*TaStb16q*		S-domain	Resistance against *Zymoseptoria tritici*	(Saintenac et al., 2021)

RLKs, receptor-like kinases; MAMPs, microbe-associated molecular patterns; ROS, reactive oxygen species; PAMP, pathogen-associated molecular pattern.

Elongation factor (EF-Tu) receptor (EFR) is another PAMP-based RLK that perceives epitopes from bacterial EF-Tu (elf18) to induce immunity against the *Agrobacterium* transformation (Zipfel et al., 2006). During the process, the PTI pathway is established through the EFR interaction with BAK1 (Schwessinger et al., 2011). In the RLKs, perception of the *Arabidopsis* danger signal peptides 1 and 2 (PEPR1 and PEPR2) stimulates the plant immunity as a DAMPs, which binds the wound-based perception of damage-associated molecular pattern peptide 1 and 2 (PEP1 and PEP2) (Huffaker et al., 2006; Yamaguchi et al., 2006). The DAMP molecules are produced at the early stages of invasion due to the wounds, microbial infection, or PAMP treatment. Similarly, other RLK complexes that consist of chitin elicitor receptor kinase 1 (CERK1) and LysM receptor-like kinase 1/5 (LYM1/5) recognize the chitin derivative of the fungal cell wall as a MAMP elicitor molecule to regulate the expression of chitin-induced defense genes (**Figure 4**; **Table 3**) (Liu et al., 2012; Cao et al., 2014). CERK1 regulates chitin-induced defense gene expression and accumulation of callose by directly interacting and phosphorylating an RLCK PBL27 (Shinya et al., 2014). PBL27 triggers defense responses by phosphorylating MAPKKK5, which activates MPK3/6 and MKK4/5 cascade (Yamada et al., 2016). The BR-signaling component CONSTITUTIVE DIFFERENTIAL GROWTH1 (CDG1) negatively regulates flg22 and chitin-triggered immunity through the interaction with FLS2 and CERK1. CDG1 overexpression causes FLS2 and CERK1 degradation, which reduces flg22 and chitin responses. Depending on its ADP-ribosyl transferase activity, the *P. syringae* effector AvrRpm1 can cause CDG1 to interact with its host target RPM1-INTERACTING PROTEIN4 (RIN4). CDG1 is capable of phosphorylating RIN4 *in vitro* at multiple sites, which was diminished in *cdg1* null plants, reducing AvrRpm1-induced allergic reaction (Yang et al., 2021b). OsCERK1 is the LysM receptor in *O. sativa*, which together with CEBiP, regulates chitin elicitor signaling in rice. A significant reduction in defense responses induced by chitin oligosaccharides was observed by knocking down OsCERK1 (**Table 3**) (Zuo et al., 2014). A novel LRR-type *Pi65* was recently cloned from *O. sativa japonica* that showed resistance against rice blast *Magnaporthe oryzae*. Decreased resistance was observed in *Pi65* knockout gene in the GY129 variety, while its overexpression in the susceptible variety LX1 enhanced resistance to rice blast (Wang et al., 2022). Program cell death is a crucial mechanism for plant immunity but could cause excessive damage to the plant if left unregulated. An S-domain RLK SPL11 cell-death suppressor 2 (SDS2) positively regulates program cell death and immunity in rice. Elevated immune responses and enhanced resistance were observed in the SDS-overexpression lines while showing susceptibility in *sds2* mutants against blast fungus *M. oryzae* (Fan et al., 2018a).

During a pathogen attack, the stress responses are regulated through the activation of cell wall integrity maintenance and immune signaling systems resulting from the cell wall damage. In *Arabidopsis*, WAK1 and WAK2 are known to maintain cell wall integrity. They do so by binding soluble oligo-galacturonide (OG) pectin and bacterial elicitor activating WAK-dependent, distinct stress-like response pathway to help plants resist a pathogen attack (Kohorn et al., 2016). Recently, several WAKs have been reported in several crop species, playing a role in immune responses against fungal and bacterial pathogens. For instance, individual OsWAKs have been reported to respond differently to rice blast fungus. OsWAK14, OsWAK91, and OsWAK92 positively regulate resistance, while OsWAK112d is a negative regulator of blast resistance. Moreover, enhanced H_2_O_2_ production required OsWAK91 to confer resistance during infection (Delteil et al., 2016). The TaWAK2 is responsible for the prevention of Fusarium head blight disease caused by *Fusarium graminearum*. TaWAK2 produces a more rigid cell wall, limiting the expression of *pectin methyl esterase 1* (Gadaleta et al., 2019). Two independent studies identified TaWAK-6D and TaWAK7D in wheat, contributing to resistance against *Fusarium pseudograminearum* and *Rhizoctonia cerealis* (Qi et al., 2021a; Qi et al., 2021b). In maize, *qHSR1* and ZmWAK-RLK1 (*Htn1*) have been reported in plant resistance against *Sporisorium reilianum* and *Exserohilum turcicum*, respectively (Zuo et al., 2015; Yang et al., 2019).

Cysteine-rich receptor-like kinases (CRKs) have the most significant number of RLKs, which are triggered during defense and oxidative stress mechanisms. CRKs are involved in program cell death and defense responses, employing both PTI and elicitor-triggered immunity (ETI) responses, in which the RLK-mediated signaling upregulates the transcriptional activation of defense (Du and Chen, 2000; Chen, 2001). For example, overexpression of CRK5 and CRK13 upregulates the immunity-related RLKs, such as pathogenesis-related protein1/5 (PR1/5) and isochorismate synthase1 (ICS1), which eventually enhance defenses against *P. syringae*. Similarly, CRK45 provides similar enhanced immunity against *P. syringae*, while *crk45* mutant displayed more susceptibility to the pathogen (Zhang et al., 2013a). CRK2 is also known to induce full elicitor-induced ROS bursts while regulating plant innate immunity. The mutant *crk2* displayed impaired defense responses against *P. syringae* (Kimura et al., 2020). CRKs, such as CRK4/5 and CRK19/20, are also known to induce cell death hypersensitive-like responses in plants (Chen et al., 2003; Chen et al., 2004; Acharya et al., 2007). CRK28-enhanced expression in *Arabidopsis* increased disease resistance to *P. syringae* while inducing cell death in *N. benthamiana*. CRK28 forms complexes with FLS2 and BAK1 independent of its kinase activity, suggesting that CRK28 kinase activity is not required during this interaction (Yadeta et al., 2016). In rice, the CRK6 and CRK10 are required for NPR1 homolog 1 (NH1)-mediated immunity (Chern et al., 2016). In *T. aestivum*, TaCRK1 provides protection against *R. cerealis* infection, while HvCRK1 of *H. vulgare* contributes to ROS-mediated basal resistance against powdery mildew infection (Rayapuram et al., 2012; Yang et al., 2013). Another wheat CRK *Stb16q* has been reported to provide resistance against *Zymoseptoria tritici*, which causes septoria tritici blotch (STB), representing one of the most genetically diverse and devastating wheat pathogens worldwide (Saintenac et al., 2021). Similar responses have also been reported in wheat by studying *Stb6*, which encodes a WAK-like (WAKL) protein to provide immunity against *Z. tritici* (Saintenac et al., 2018). A novel wheat TaCRK2 positively regulates resistance against leaf rust, caused by *Puccinia triticina* in a Ca^2+^-dependent manner (Gu et al., 2020). Another study found TaCRK3 having two DUF26, providing defense against *R. cerealis* mycelia (Guo et al., 2021). Similarly, wheat TaCRK10, similar in function to TaXa21, provides resistance against stripe rust under high temperatures. While *TaCRK10* and *TaXa21* share a similar gene function, their molecular targets differ, suggesting that plants have developed distinct defense mechanisms against the same pathogen. This could work to their benefit if the pathogen suppresses one of the pathways (**Table 3**) (Wang et al., 2021a). It has been reported that OsRMC, which has DUF26 as an extracellular domain, plays a role in the development of the salt stress response and rice root meander curling (Jiang et al., 2007; Zhang et al., 2009). In *Medicago truncatula*, the early senescence and defense responses are prevented by SymCRK associated with symbiotic interactions (Berrabah et al., 2014).

In *Arabidopsis*, SOBIR1 was identified through genetic screening of the suppressors of *bir1-1*, which showed excessive cell death in mutant form. *sobir1* mutant, on the contrary, showed rescued cell death, suggesting that SOBIR1 is a positive regulator of cell death (Gao et al., 2009). Similarly, the *Arabidopsis* SOBIR1 and kinase-active BAK1 together constitutively activate immune responses through autophosphorylation and transphosphorylation events (Van der Burgh et al., 2019). A recent study reported that the phosphorylation of Thr529 and activation of β3-αC loop are essential for SOBIR1 activation by BAK1 transphosphorylation during SOBIR1-induced cell death response in *N. benthamiana* (Wei et al., 2022). In rice, immunity against rice black-streaked dwarf virus (RBSDV) infection is mediated by OsSOBIR1 interaction with OsRLP1, triggering PTI responses (Zhang et al., 2021a). The NIK1 is an LRR-type RLK, which was previously found to participate in antiviral immunity by translocation of RLP10 into the nucleus. The RLP10 downregulates the translation machinery by making a complex with LIMYB, resulting in host and viral mRNA inhibition and tolerance against *Begomovirus* (Machado et al., 2015). Another study reported that NIK1 also acts as a negative regulator of antibacterial immunity. In addition, it also negatively regulates the FLS2-BAK1 complexes. This unique inverse modulation can allow viruses and bacteria to use host immunity against each other (**Table 3**) (Li et al., 2019a).

During plant growth and development, several environmental and other related factors can lead to the plant’s abiotic stresses. Plants in such conditions use sophisticated mechanisms through utilizing diversified RLKs. In plants, the osmotic imbalance is caused by drought and salinity. It is well recognized that ABA affects osmotic balance and stomatal closure during abiotic stress responses (Zhang et al., 2020). Some CRKs are found to regulate ABA signaling and its biosynthesis. CRK4, CRK5, and CRK19 redundantly confer ABA responses, enhancing drought tolerance (Lu et al., 2016). Similarly, the expression of ABA biosynthesis genes, including abscisic aldehyde oxidase 3 (AAO3), ABA deficient (ABA1 and ABA2), and 9-*cis*-epoxycarotenoid dioxygenases (NCED3 and NCED5), are positively regulated by cytoplasm-localized CRK45. This increases tolerance to drought and salt stress during germination and post-germination growth (Zhang et al., 2013b). In addition to CRKs, the LRR-type HSL3 also plays a role in tolerance to drought by regulating the stomatal closure. It has been demonstrated that HSL3 can adjust the amount of H_2_O_2_ in the guard cells, hence negatively regulating stomatal closure. Dehydration or treatment with ABA or H_2_O_2_ greatly upregulated HSL3 (Liu et al., 2020b). *Arabidopsis* RLK7 is associated with stress and immune responses that interact with phytophthora-inhibited protease 1 (PIP1), activating S-type anion channel SLAC1 to induce stomatal closer (Pitorre et al., 2010; Shen et al., 2020). The plasma membrane-localized PdERECTA reduced stomatal density and limited water-consuming cells, restricting water consumption and enhancing drought resistance in poplar (Li et al., 2021a). A similar study showed that the expression of *S. bicolor SbERECTA* (*SbER*) in *Arabidopsis* and maize confers enhanced drought tolerance (Li et al., 2019c). More recently, it was discovered that BAK1 can activate H+-ATPase isoform 2 (AHA2) by phosphorylating its C-terminal Ser944, which causes guard cells’ cytoplasm to become alkaline, initiating the process of stomatal closure (Pei et al., 2022).

A number of RLKs have been shown to be involved in other stress responses such as salt, metal, and temperature stresses. For instance, WAK1 of the WAK family is implicated in responses against aluminum toxicity in *Arabidopsis* and rice beans. The AtWAK1 overexpression showed increased tolerance to aluminum stress in *Arabidopsis* (Sivaguru et al., 2003; Lou et al., 2020). The rice OsWAK11 has been implicated in copper, aluminum, and sodium responses. OsWAK11 detoxified excessive copper and knocked it down, resulting in hypersensitivity to copper toxicity (Hu et al., 2014; Xia et al., 2018). OsSIT1, a lectin-type RLK, is known to negatively regulate resistance to salt stress. OsSIT1 phosphorylates the downstream effectors MPK3 and MPK6, causing the production of ethylene and salt-induced ethylene signaling (Li et al., 2014a). Another rice RLK, OsSTLK, was implicated in salt tolerance with improved salt stress tolerance and reduced salt sensitivity observed in OsSTLK overexpression rice lines (**Table 3**) (Lin et al., 2020). Similarly, a cold-inducible gene, *GsLRPK*, from *Glycine soja* enhances resistance to cold stress and increases the expression of a number of cold-responsive genes when expressed in yeast and *Arabidopsis* (Yang et al., 2014). Another two cold-tolerance genes, *MtCTLK1* and *MfCTLK1* from *M. truncatula* and *Medicago falcata*, respectively, are also known to regulate cold tolerance (Geng et al., 2021).

## 6 The role of receptor-like cytoplasmic kinases

RLCKs play a crucial role in RLK-mediated signaling. The N-myristoylation or palmitoylation enables most RLCKs to localize on the plasma membrane (Lin et al., 2013). A combined phylogeny with RLKs conducted in *Arabidopsis* and rice showed that there are 17 subgroups of RLCKs including subgroups II and IV to XIX (Shiu et al., 2004). Most of the RLCKs consist of Ser/Thr-based KDs, while others possess additional domains such as LRR, EGF, and TM (Vij et al., 2008; Lehti-Shiu et al., 2009; Lin et al., 2013). RLCKs regulate several functions such as hormone signaling, sexual reproduction, shoot and root meristem maintenance, stomatal patterning, petal abscission, differentiation of vascular tissues, innate immunity, and adaptation to abiotic stresses in a coordinated manner with RLKs and RLPs (Lin et al., 2013; Tang et al., 2017; Liang and Zhou, 2018). For example, during the immune responses, the BIK1 and other associated RLCKs directly interact with various immune-related RLKs (Lu et al., 2010; Liu et al., 2013; Kong et al., 2016). Similarly, BR-induced signaling is regulated via direct interaction of RLCK-XII subclass BR signaling kinases (BSKs) with BRI1 to control different subsequent processes (Tang et al., 2008; Sreeramulu et al., 2013). In *Arabidopsis*, the pollen tube integrity is controlled through the downstream action of RLCK MARIS (MRI) from the two pairs of closely related receptors, buddha’s paper seal (BUPS1) and BUPS2 and ANXUR1 (ANX1) and ANX2 (**Figure 3**) (Boisson-Dernier et al., 2015; Liao et al., 2016; Ge et al., 2017). In *Brassica*, the self-incompatibility is regulated through the interaction of an RLCK M-locus protein kinase (MLPK) with S-locus b-lectin receptor kinase (SRK), a female determinant (Kakita et al., 2007). The RLCKs’ primary regulatory mechanism of RLK-mediated signaling is controlled through the transphosphorylation of RLKs and different downstream substrates where the RLCKs activity and stability are firmly regulated (Sreeramulu et al., 2013; Li et al., 2014b; Nolan et al., 2017; Wang et al., 2018).

**Figure 5:**
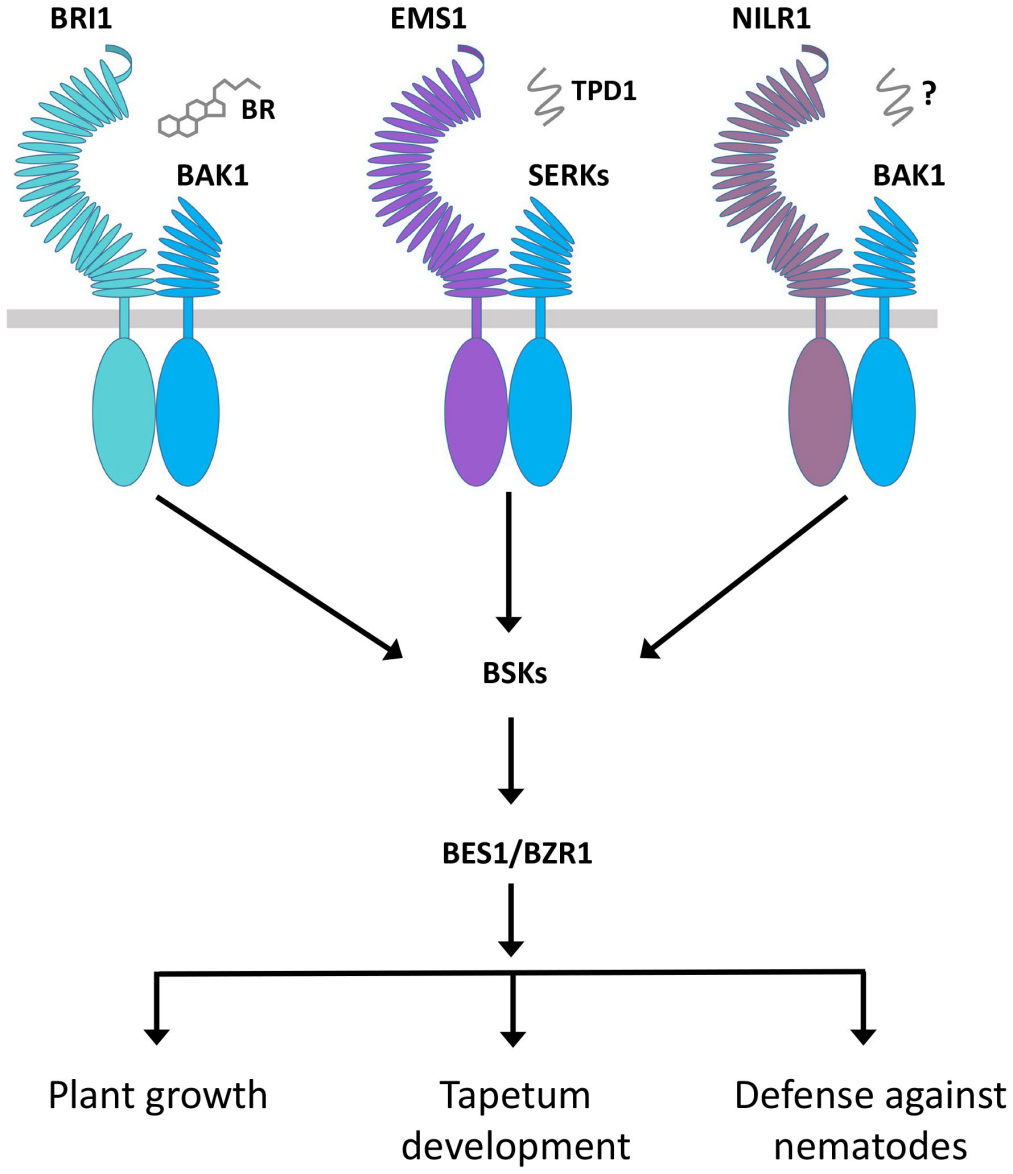
Regulation of diverse functions of BRI1, EMS1, and NILR1 through the same signaling pathway. BRI1 and EMS1 perceive BR hormone and TPD1 peptide ligand, respectively, while NILR1 binds an unknown ligand to collectively initiate a pan-brassinosteroid signaling cascade to control their distinct functions.

In RLK-mediated signaling, the RLK KD determines the signal output for specificity. In this case, different kinds of RLCKs are associated with a variety of RLKs. In several cases, different RLKs can associate the same RLCK to regulate distinct functions. For instance, both BRI1 and FLS2 interact with BIK1. The BRI1-BIK1 interaction leads to the negative regulation of root hair, while the FLS2-BIK1 interaction is responsible for the positive regulation of immunity (Veronese et al., 2006; Lu et al., 2010). Similarly, the BSKs can interact with BRI1, EMS1, NILR1, and FLS2 to positively regulate plant growth, tapetum formation, and immunity (Shi et al., 2013; Zheng et al., 2019; Zheng et al., 2022a). In the case of EMS1, BRI1, and NILR1, they all may bind distinct ligands to control their different functions but use the same signaling components, such as BSKs, BKI1, BIN2, and BES1/BZR1, in the same signaling pathway, which opens up a new insight into the regulation of RLK signaling (**Figure 5**; **Table 2**) (Zheng et al., 2019; Zheng et al., 2022a). All these studies suggest that the fate of RLCK is determined by ligand-based upstream RLK complexes, but the exact mechanism is still unknown. The RLCKs interpret different signal outputs differently and give rise to distinct ones. BSKs are the direct substrates of BRI1 and are directly phosphorylated by BRI1. Similarly, BSK1 also interacts with and is phosphorylated by NILR1 and FLS2. The phosphorylation of BSK1 by BRI1 takes place in a similar manner to that of FLS2 and NILR1, but this interaction can only induce plant growth but not the immune responses (Shi et al., 2013; Zheng et al., 2022a). It is possible that the BSK1 distinct output signal is caused by the differential phosphorylation. In addition, other signaling components and complexes as well as environmental cues may also play a role in defining the function of RLCKs.

## 7 LRR-RLKs: the biggest class of RLKs

In *Arabidopsis*, the LRR-RLK subgroup consists of approximately 200 members, making it the largest class of RLKs (Shiu and Bleecker, 2001a). The KD of LRR-RLKs is conserved among the group members, but the ECD that possesses variable numbers and organization of LRRs ranging from 3 to 31, dependent on the kind and the subgroup of LRR, is highly versatile (Pfister et al., 2014; Hohmann et al., 2017). The LRR-RLKs have been studied extensively over the past two decades. Still, due to the large number of RLKs within the group, the number of designated members is approximately 89, and only 60 of them have been characterized functionally, making up 35% of the total LRR-RLKs in *Arabidopsis* (Wu et al., 2016). Based on experimental studies, these characterized LRR-RLKs can be classified as either plant growth and development regulators or involved in plant defense and immunity (He et al., 2018; Jamieson et al., 2018). Many LRR-RLKs that have been identified were previously misclassified into other gene families (Man et al., 2020). In *Arabidopsis*, the LRR-RLKs are further divided into 15 subclasses based on their KD conservation (Shiu and Bleecker, 2001a). The LRR is composed of 24 amino acid residues with consensus sequence LxxLxLxxNxLxxGxIPxx, where x represents non-conserved residues (Torii, 2004). In plants, this unique motif of 24 amino acid residues forms the helical horseshoe-like structure characterized by having β-turn or β-sheet acting as a protein–protein interaction site (Kobe and Deisenhofer, 1993; Jones and Jones, 1997). Interestingly, this signature horseshoe-like structure is not common to all LRR-RLKs, but instead, some of the LRRs have a right-handed super-helical structure, such as BRI1, which is different than the solenoid conformation (Hothorn et al., 2011; She et al., 2011). The GxIP sequence configuration forms the helical structure and is common to many LRRs such as FLS2, BRL1, and RPK2 (She et al., 2013; Sun et al., 2013b; Song et al., 2014). There is a comparable size difference among distinct LRR-RLKs, which vary from 19 to 21 LRRs. The super-helical structure is formed by approximately 20–25 LRRs, where the lateral and inner surface of the structure actively achieves its binding with other protein molecules (Sun et al., 2013a; Sun et al., 2013b). Some of the LRR-RLKs with different numbers of LRRs include BRI1 25 LRRs, PSKR1 21 LRRs, GSO1 31 LRRs, FLS2 29 LRRs, HAE 21 LRR, TMK1 13 LRRs, PGIP 10 LRRs, RPK2 22 LRRs, and SERK1 5 LRRs (Pfister et al., 2014; Hohmann et al., 2017). Several studies have suggested that the size of LRR-RLK can be exploited to uncover the function of LRR-RLK as a receptor or coreceptor (Jaillais et al., 2011; Smakowska-Luzan et al., 2018). The small-sized LRRs are generally considered as coreceptor; for example, CIKs and SERKs have been identified with shorter ECD serving as coreceptors for the protein–protein interaction.

In the regular region of LRRs, a spacer ID is identified between LRR21 and LRR22 (corresponding to 584–654 residues), making them the fourth to fifth LRRs from the C-terminus. In BRI1, this 70 amino acid residue segment folds back into the interior of the super-helix, forming a small domain that generates an interaction site for the BR (Li and Chory, 1997; Hothorn et al., 2011). In addition, the crystal structure of RPK2 revealed two ID regions where the ligand binds (Song et al., 2014). Other LRR-RLKs containing an ID include TMK1, PSKR1, and PSY1R (Matsushima and Miyashita, 2012). Some studies have also reported another interesting domain called Cys-pair in the ECD. The Cys-pair is located at the N-terminus near the start codon between the signaling peptide (SP) and the first LRR. In some cases, a second Cys-pair is also present between the TM and the last LRR (Diévart and Clark, 2003). The mutation in Cys-pair does not affect the function in CLV2 (Song et al., 2010), but in some LRR-RLKs, the Cys-pair mutation affects the receptor’s normal function. For example, FLS2 with Cys-pair mutation showed a significant decrease in its activity (Sun et al., 2012). In BRI1, the *bri1-5* mutant harboring Cys-pair mutation results in the retention of the *bri1* mutant in the endoplasmic reticulum, which suggests that this mutant is not able to pass the endoplasmic reticulum quality control (ERQC) to translocate into the plasma membrane and is eventually degraded by endoplasmic reticulum-associated degradation (ERAD) (Hong et al., 2008). Thus, LRRs, together with other domains in the ECD, are the essential components of the receptor and play a vital role in ligand perception, protein–protein interaction, and signal transduction.

## 8 Conclusion and perspectives

EPKs control various biological functions through the utilization of ATP, where a γ-phosphate is transferred to the free OH-site of serine/threonine or tyrosine; thus, EPKs are widely considered dynamic molecular switches (Taylor et al., 2012; Zhang et al., 2015). RLKs belong to the EPKs that resemble animal RTKs in their domain organization and play a central role in plant growth and development, reproduction, adaptation to new environmental conditions, and immune responses. The main kinase core of RLKs is highly conserved containing different domains with unique signatures and functions. The evidence of the first RLKs comes from common ancestor green algae, which are vastly duplicated and expanded in angiosperm, implying their significance in plant growth and survival during evolution. Although the exact pattern of RLK evolution is only partially resolved, the sequenced genomes of basal Streptophyte species paved the road to describe the precise evolutionary history. The expansion of RLKs might directly be related to the morphological complexity of the plant species. For example, *C. braunii* and *K. flaccidum* are Charophytes with 435 and 94 RLKs, respectively (**Table 1**), but domain expansion of LyM, CrRLK1L, and S-domain was only specific to *C. braunii*, suggesting the association of these domains with morphological complexity of a specific lineage (Dievart et al., 2020). Similarly, the ever-changing environments also play a vital role in RLK evolution where their ECD architectures are diversified to enable RLKs to perceive numerous environmental cues and respond accordingly. The intracellular KD remains conserved but evolves enough changes that can recruit different complexes to execute the signals in response to those environmental cues. Gaining an in-depth understanding of RLK families, especially the expansion of the large families, such as LRR, PERK, and Lec, the ancestral and recent lineage-specific duplication, or domain rearrangement events must be taken into account, which makes it a challenging task.

Among the vast number of RLKs, only a handful have been identified and functionally characterized through genetic studies. Plants rely on an RLK‐mediated signal transduction pathway that utilizes intracellular regulatory components to alter gene expression profiles, in response to an extracellular signal. RLKs regulate a vast array of gene functions during plant growth and development. The detailed molecular mechanism of the functionally characterized gene is still not completely defined. Several RLKs regulate multiple developmental processes. For example, HAE and HSL2 play a dual role in regulating cell separation during floral organ abscission and lateral root development (Jinn et al., 2000; Zhu et al., 2019). ACR4 regulates not only root apical meristem but also sepal development, integument development, and epidermal development (Gifford et al., 2003; Tanaka et al., 2007; De Smet et al., 2008). GSO1/2 regulates normal embryo development and root growth development (Tsuwamoto et al., 2008; Racolta et al., 2014). For future studies, it would be interesting to ask how a single RLK can regulate multiple functions or how different RLKs interact with each other to ensure the same functional regulation.

Till recently, traditionally, it was believed that RLKs use unique signaling pathways to control their distinct functions. The first evidence of multiple RLKs utilizing the same signaling cascade to control diverse functions emerged recently, showing that BRI1, EMS1, and NILR1 bind entirely different ligands to control their unique functions; however, they utilize the same pan-brassinosteroid signaling cascade (**Figure 5**) (Zheng et al., 2019; Zheng et al., 2022a). Studies like this entirely changed the perspective of the RLK signaling mechanism. Techniques such as exchange domain, proteomics-based RLK mining with different genetic backgrounds, single-cell-omic analysis, and cellular and subcellular imaging may help to understand RLK signaling pathways and networks at a higher precision.

One of the most important and challenging tasks regarding the RLK signaling mechanism is the identification of ligands. So far, only a limited number of RLK-binding ligands have been identified, and the majority of RLK-binding ligands are unknown. Novel techniques are expected to be developed, which can be employed together with the existing technologies of proteomic analyses, genetic approaches, structural biology, and photoaffinity crosslinking technology for ligand identification.

As the RLKs are extensively duplicated and expanded in plants, another challenge comes from the functional redundancy of genes, which makes it difficult to identify the functions of RLKs. For example, the BAK1 interacts with BRI1 as a coreceptor to regulate BR-dependent growth promotion. BAK1 also regulates FLS2 and EFR signaling pathways to induce immune responses. Similarly, a double knockout mutant *gso1gso2* is required to study the function of GSO1 and GSO2 that interact with two ligand peptides CIF1 and CIF2 (Nakayama et al., 2017). However, advanced techniques, like CRISPR for generating high-order RLK mutants and advanced method phenotypic analysis, will help overcome functional redundancy to understand their roles in the future.

Signaling pathway crosstalk is a common mechanism in plants. One RLK might be associated with multiple pathways apart from the already known ones as in the case of BAK1. The most interesting question about RLKs is how these RLKs define their functions, especially when an opposite output produces from each other if they use the same signaling components or even the same signaling pathways. Technologies such as genetics, structural, and proteomics will play a vital role in defining all these questions. These studies will play a significant role in improving crop yield and more resistant cultivars to biotic and abiotic stresses via bioengineered and plant breeding approaches.

## Author contributions

JL: Software, Writing – review & editing. WL: Investigation, Writing – review & editing. GW: Funding acquisition, Validation, Writing – review & editing. KA: Conceptualization, Writing – original draft, Software.
